# Optimization of Controlled-Release Microspheres Containing Vitexin and Isovitexin Through Experimental Design and Evaluation of Their Hypoglycemic Effects

**DOI:** 10.3390/pharmaceutics17070819

**Published:** 2025-06-24

**Authors:** Nhu Huynh Mai, Hoang-Han Do, Phi Hoang Yen Tran, Cong-Phi Nguyen, Van-Ha Nguyen, Ngoc Phuc Nguyen Nguyen, Kien-Duc Ngo, Duc-Tuan Nguyen, Minh-Quan Le

**Affiliations:** 1 Department of Pharmacology, University of Medicine and Pharmacy at Ho Chi Minh City, Ho Chi Minh City 700000, Vietnam; mhnhu@ump.edu.vn (N.H.M.); nnpnguyen.d21@ump.edu.vn (N.P.N.N.); 2 Department of Pharmaceutical Technology, University of Medicine and Pharmacy at Ho Chi Minh City, Ho Chi Minh City 700000, Vietnam; dhhan@ump.edu.vn (H.-H.D.); yen.tranphihoang@ump.edu.vn (P.H.Y.T.); ncongphi@ump.edu.vn (C.-P.N.); nguyenvanha@ump.edu.vn (V.-H.N.); 3 Department of Biochemistry, University of Medicine and Pharmacy at Ho Chi Minh City, Ho Chi Minh City 700000, Vietnam; ngokienduc@ump.edu.vn; 4 Department of Analytical Chemistry—Drug Quality Control, University of Medicine and Pharmacy at Ho Chi Minh City, Ho Chi Minh City 700000, Vietnam; ductuan@ump.edu.vn

**Keywords:** microspheres, controlled release, hypoglycemic effects, isovitexin, vitexin

## Abstract

**Background/Objectives**: Vitexin and isovitexin are bioactive flavonoids with promising pharmacological effects; however, they have poor bioavailability. Microencapsulation with biodegradable polymers is a promising strategy for improving their stability, bioavailability, and biocompatibility. This study aimed to optimize the formulation parameters to obtain microspheres with desired properties in terms of size, loading ratio, and vitexin–isovitexin release. **Methods**: Microspheres were prepared using alginate as the core matrix and a chitosan outer layer. A Design of Experiment approach using response surface methodology was employed. The hypoglycemic effects of the obtained microspheres were evaluated. **Results**: The formulation using 1.17% low-viscosity alginate, 7.60% calcium chloride, 5.78% Tween 80, and 5.00% Span 80 resulted in microspheres with optimal mean size (10.78 µm), high loading ratio (22.45%) and encapsulation efficiency (68.92%). The in vitro release of vitexin–isovitexin from microspheres was completed within 24 h in controlled manner. The microspheres were found to be non-toxic in vivo and exhibited hypoglycemic effects after 21 days at doses equivalent to 30 and 60 mg/kg of vitexin–isovitexin. The potential mechanisms might involve increasing the size of Islets of Langerhans and improving pancreatic β-cell function and insulin resistance, as observed in alloxan-induced diabetic mice. **Conclusions**: This work successfully developed alginate–chitosan-based microspheres for the controlled release of vitexin–isovitexin while maintaining their bioactivities.

## 1. Introduction

Diabetes mellitus has claimed its position among the top burden health disorders of the century owing to its emerging increase in incidence and complex pathophysiological progress [1]. Searching for new antidiabetic chemical entities of both synthetic and natural origin has therefore attracted much scientific interest and has eventually resulted in plentiful breakthroughs. Studies on natural products have indicated that medicinal plants and their isolated compounds can be successfully used for the management of diabetes mellitus [2,3]. Amongst these significant findings, vitexin and isovitexin, two of the most published antidiabetic flavonoids, have recently received increasing attention due to their pharmacological relevance in diabetes mellitus [2,4,5].

**Figure 1:**
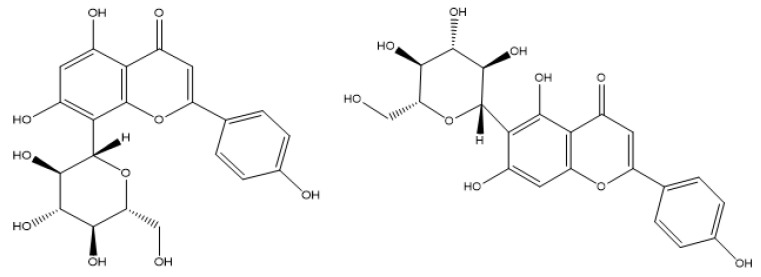
The structure of vitexin (**left**) and isovitexin (**right**).

Vitexin and isovitexin are flavones, a major subclass of natural flavonoids, commonly isolated together in a mixture. These two compounds are isomers of each other, as they are mono-*C*-glycosylated derivatives of apigenin. While vitexin is characterized as an apigenin-8-*C*-glucoside, the glucosyl moiety of isovitexin binds to the aglycone through a C-C linkage at the C6 atom (shown in Figure 1). Hence, these two compounds share almost all chemical properties [3,6]. Compared to the aglycone apigenin and its other *O*-glycosylated derivatives, vitexin and isovitexin exhibit higher stability, and antioxidant and antidiabetic effects in most cases [7]. Several reports indicated that vitexin has had a wide range of pharmacological effects on a systemic scale, including antioxidant, anti-inflammatory [8], anti-Alzheimer’s, and antidiabetic effects [5,9,10]. Isovitexin has health-protecting effects similar to those of vitexin, as its structural resemblance has been thoroughly discussed [5,10].

In terms of their protective capacity against diabetes, vitexin and isovitexin act through multiple mechanisms in multiple targets [2,11], especially enzymes and pancreatic β-cells. Vitexin and isovitexin inhibit α-amylase by reducing its activity. In contrast, the reduction in α-amylase activity of vitexin was higher affinity and more stable than that of isovitexin [12]. In addition, vitexin and isovitexin have obvious effects in decreasing blood glucose and inhibitory effects on α-glucosidase as natural antihyperglycemic agents [10]. Other in vitro studies reported that both vitexin and isovitexin inhibit aldose reductase activity [13,14,15]. An in vivo study using orally administered rats demonstrated that both vitexin and isovitexin significantly reduced postprandial blood glucose concentrations in a dose-dependent manner [10,16]. Several toxicity studies have demonstrated the safety of vitexin. To date, in vitro and in vivo studies have focused on the safety of vitexin but very rarely on isovitexin. Purified plant extracts containing high concentrations of vitexin and isovitexin showed no significant acute or subchronic toxicity or genotoxicity [9,17].

Unfortunately, vitexin and isovitexin are poorly absorbed in the digestive tract [18], indicating that they possess low oral bioavailability [19] and are barely degradable by normal digestive enzymes. In the case of the intravenous route, vitexin and isovitexin are widely distributed in different tissues, with high concentrations in the liver and kidneys [19,20,21,22]. The half-life of orally administered vitexin in rats is approximately 6.3 h, while the intravenous route has T_1/2_ of less than 1 h [4,19,23]. Decades of research have suggested that vitexin and isovitexin are strong candidates for further drug discovery studies and subsequent clinical trials. However, it is still a major challenge to enhance the efficacy of vitexin and isovitexin, which is necessary for designing a suitable drug delivery system to protect against degradation and control the release of substances.

To overcome these challenges, drug delivery systems containing vitexin and isovitexin have been developed. It has been demonstrated through studies that several drug delivery systems, including liposomes [24], microemulsions [25], microspheres from β-cyclodextrin [26], nanoparticles [27,28], and microparticles [29] may efficiently increase solubility and regulate release in vitro. Studies that concentrate on either pure vitexin or a combination of other compounds found in medicinal herbs are becoming more common, but there are not many that consider both vitexin and isovitexin at the same time. However, there is no published paper utilizing alginate–chitosan microspheres as carriers of these two compounds.

Microspheres refer to capsules of a spherical shape measured in microns and with diameters usually in the range of approximately 1–1000 µm [30]. Encapsulation creates a physical barrier that protects substances from environmental conditions and improves their bioavailability through a controlled-release profile. This indicates that it is suitable for encapsulating vitexin and isovitexin to form microspheres. Biodegradable polymers are commonly used to prepare microspheres because of their stability, reduced volatility, release characteristics, and environmental compatibility [31]. To produce an optimal carrier system for hydrophobic compounds, such as vitexin and isovitexin, sodium alginate and chitosan were chosen to prepare alginate–chitosan microspheres by ionic cross-linking to form polyelectrolyte complexes’ (PECs) microspheres.

To the best of our knowledge, no study has been conducted on the encapsulation of a mixture of vitexin and isovitexin in alginate–chitosan microspheres. Thus, this study was designed to optimize the formulation parameters to achieve vitexin–isovitexin-loaded microspheres with optimal particle mean size, loading capacity, encapsulation efficiency, and the ability to control the compounds’ release. In vivo bioaccessibility studies were carried out to determine the biocompatibility of the obtained microspheres on mice and their hypoglycemic effects. To date, several animal models of diabetes have been used to evaluate the potential of novel antidiabetic medications, including high-fat diet [32,33], streptozotocin [34], alloxan [35], and fructose-fed [36,37]. Alloxan and streptozotocin are the most frequently used diabetogenic agents because of their capacity to simulate hyperglycemic conditions for 2–4 weeks in animals. In terms of affordability, alloxan is considered more available and cost-effective than streptozotocin [38]. Owing to the advantages of the alloxan model, it was chosen for hyperglycemia induction in the in vivo experiment in our research. This study provides a new promising drug delivery system to load hydrophobic compounds and control their release to enhance bioavailability, leading to the investigation of their release kinetics and evaluation of their acute toxicity and hypoglycemic effects in animal models.

## 2. Materials and Methods

### 2.1. Materials

A purified (>95%) compound of vitexin and isovitexin extracted from *Mung bean* seed coat, with a vitexin–isovitexin ratio of 1:1, was provided by Napro (Hanoi, Vietnam). Three types of alginates with different viscosities were purchased from TCI (Tokyo, Japan): low viscosity (100–200 cps of 1% solution), medium viscosity (300–400 cps), and high viscosity (500–600 cps). High-molecular-weight chitosan was purchased from Sigma-Aldrich (Taufkirchen, Germany). Other chemical agents used in the preparation, including isooctane, Tween 80, Span 80, calcium chloride, acetone, and acetic acid, were provided by Fisher Scientific (Bremen, Germany). HPLC analysis agents, such as phosphate buffer and acetonitrile, were purchased from Fisher Scientific, and orthophosphoric acid 85% was bought from VWR Chemicals (Pennsylvania, PA, USA) [39]. Alloxan monohydrate (A7413-25g) was obtained from Sigma-Aldrich [39]. α-glucosidase enzyme from *Saccharomyces cerevisiae* (12.74 U/mg, lot No.: 118M4045V, Sigma-Aldrich), p-NPG, and acarbose (Sigma-Aldrich) were purchased.

### 2.2. Preparation of Vitexin–Isovitexin-Loaded Microspheres

This process was divided into two stages: synthesis of alginate cores using the water-in-oil (W/O) emulsion technique, followed by external gelation and coating of chitosan onto the alginate core surface to form microspheres.

Based on our previous studies [40,41], the alginate cores were prepared via ion gelation through the emulsification stage. The determined alginate types and concentrations were dissolved in distilled water and magnetically stirred overnight. The vitexin–isovitexin mixture (200 mg) was dispersed and mixed completely to form a homogenizer suspension with alginate. The alginate suspension was then gently added to 40 g of isooctane containing Span 80 using a homogenizer (IKA T25, Digital Ultra-Turrax, Staufen, Germany) at 7200 rpm to create a W/O emulsion. After 5 min, the Tween 80 solution was added to the W/O emulsion under homogeneous conditions for 3 min, a calcium chloride solution was added, and the mixture was stirred for 15 min. The alginate-based microspheres were formed and solidified in acetone for 10 min under magnetic stirring. Then, centrifugation was performed at 4000 rpm for 30 min, followed by vacuum filtration using a 0.45 µm nylon filter to obtain alginate-based microspheres that were triple-washed with acetone and dried at room temperature. The alginate-based microspheres were stored under proper conditions before evaluation in the subsequent stages.

The chitosan encapsulation process using the immersion method included the following steps: High-molecular-weight chitosan at a concentration of 0.5% (*w*/*w*) was dissolved in 1% (*w*/*w*) acetic acid to form a polymer solution, which was then adjusted to pH 5 using a 1 M sodium hydroxyl solution. The optimal alginate cores were immersed in a chitosan polymer solution with magnetic stirring at approximately 1000 rpm for 2 h at room temperature. The ratio between the alginate cores and the chitosan solution was 1:100. The microspheres were recovered by centrifugation at 4000 rpm for 5 min, followed by vacuum filtration through a 0.45 µm cellulose acetate filter membrane. Subsequently, the microspheres were washed several times with distilled water to remove excess chitosan on the surface and dried at 37 °C. The microspheres were stored in sealed bottles in a dry environment for the subsequent research.

### 2.3. Characterization of the Microspheres

#### 2.3.1. Morphology of Microspheres

The morphologies of the alginate cores and alginate–chitosan microspheres prepared under optimal conditions were examined by scanning electron microscopy (SEM) (JEOL-JSM–6400, Tokyo, Japan). SEM photographs were captured at different magnifications using an acceleration voltage of 10.0 kV, and the interfacial shape and porosity of the microspheres were examined.

#### 2.3.2. Zeta Potential and Differential Scanning Calorimetry (DSC)

The optimal microspheres were analyzed using the zeta potential on a Zetasizer ZEN 3600 (Mavern Panalytical, Malvern, UK) to determine the surface charge and steric stability of the alginate core structure during storage.

Differential scanning calorimetry analysis was performed using a DSC 25 (TA Instruments, New Castle, DE, USA) under a nitrogen atmosphere to examine the possible transformation interactions in the optimized alginate core and the final microsphere. Approximately 5 mg of microspheres was sealed in aluminum pans and heated at 5 °C/min from 50 to 300 °C. Structural changes in the optimized alginate core and the final microsphere were recorded as the temperature increased during the test cycle.

#### 2.3.3. Evaluation of Microsphere Size

The microsphere size distribution and mean particle size were determined experimentally using a laser scattering particle size distribution analyzer LA-920 (Horiba, Kyoto, Japan). Small amounts of microspheres were slowly added to the measuring cup until the concentration reached the required range, and the results were recorded. The measurement was repeated three times, and the average value was calculated.

#### 2.3.4. Evaluation of Vitexin–Isovitexin Loading Capacity and Encapsulation Efficiency

A method for the simultaneous quantification of vitexin and isovitexin in microspheres using high-performance liquid chromatography (Nexera XR, Shimadzu, Kyoto, Japan) with a PDA detector was developed and validated. The mobile phase of (A) acetonitrile, and (B) 0.1% orthophosphoric acid with the gradient procedure started with 15% (A) and decreased to 10% after 15 min; the mobile phase (A) was maintained at 15% until 40 min. The other chromatographic conditions were a column temperature of 30 °C, a detection wavelength of 335 nm, 20 μL of injection volume, and flow rate of 1.0 mL/min. This method was used to determine the vitexin and isovitexin loading capacities and encapsulation efficiencies of the microspheres.

An accurate mass (10.0 mg) of the microspheres was suspended in 100 mL phosphate buffer (pH 7.4) and stirred at room temperature until the microspheres completely released the active ingredient. The vitexin–isovitexin-loaded microspheres were analyzed by HPLC. Each sample was quantified in triplicate, and the average value was calculated. The loading capacity (LC) (1) and encapsulation efficiency (EE%) (2) were calculated as follows:

(1) $$\mathrm{LC}\%=\frac{\mathrm{amount}\;\mathrm{of}\;\mathrm{vitexin-isovitexin}\;\mathrm{in}\;\mathrm{microspheres}\;(\mathrm{mg})}{\mathrm{amount}\;\mathrm{of}\;\mathrm{microspheres}\;(\mathrm{mg})}\;\times \;100$$

(2) $$\mathrm{EE}\%=\frac{\mathrm{amount}\;\mathrm{of}\;\mathrm{vitexin-isovitexin}\;\mathrm{in}\;\mathrm{microspheres}\;(\mathrm{mg})}{\mathrm{amount}\;\mathrm{of}\;\mathrm{vitexin-isovitexin}\;\mathrm{in}\;\mathrm{theory}\;(\mathrm{mg})}\;\times \;100$$

#### 2.3.5. In Vitro Release and Kinetics Studies

Approximately 20.0 mg of microspheres was suspended in a 100 mL PBS pH 7.4 solution and maintained at 37 °C under stirring at 100 rpm. Samples (5 mL) were collected from the release medium every hour and replaced with the same volume of fresh medium. The absorbance of the aliquots was analyzed using HPLC. All experiments were performed in triplicate, and the percentage of the cumulative amount of released vitexin–isovitexin was calculated against the time.

To investigate the kinetic model of release from the microspheres, the release data were analyzed using the following mathematical models: zero-order kinetics, first-order kinetics, Higuchi equation, Hixson–Crowell equation, and Korsmeyer–Peppas equation.

#### 2.3.6. Optimization of Alginate Cores’ Formulation

To optimize the parameters for the formulation of alginate–chitosan microspheres, Design Expert (v.13.0) software (Stat-Ease, Inc., Minneapolis, MN, USA) with I-optimal response surface methodology was used to determine the relationship between the two variables and responses. Alginate cores were prepared to optimize the vitexin–isovitexin loading capacity and ensure the relevant mechanical properties. It is vital to identify the formulation parameters that affect the properties of alginate cores. Five independent variables were optimized: (X_1_) alginate concentration, (X_2_) calcium chloride concentration, (X_3_) Tween 80 (stabilizer) ratio, (X_4_) Span 80 (emulsifier) ratio, and (X_5_) alginate type with upper and lower levels (Table 1).

The four dependent variables were chosen, namely, (Y_1_) alginate core size, (Y_2_) loading capacity, (Y_3_) encapsulation efficiency, and (Y_4_) the release rate after 1 h. Multiple regression models (e.g., No Transform, Square Root, Natural Log, Inverse Square Root, Inverse, and Power) were used to correlate the factors and response interaction and predict the optimal formulation. All the data analyzed were shown as the mean. One-way analysis of variance (ANOVA) and a *t*-test were used to test the statistical significance, with the significance determined at a level of *p* = 0.05. From there, the most suitable algorithm would be chosen by corresponding to the smallest *p*-value (with *p* < 0.05), and the largest F and R^2^ values. Using an appropriate method, the impact of each variable Xi on each output attribute Yi of the microspheres was examined in detail, and the associated coefficient *p* was calculated to determine the level of influence.

### 2.4. Acute Toxicity and Hypoglycemic Effects of Vitexin–Isovitexin-Loaded Microspheres in Animal Model

#### 2.4.1. Animals

Male Swiss albino mice, with a mean weight 20.2 ± 2 g, were obtained from the Pasteur Institute in Ho Chi Minh City. The mice showed no deformities or abnormal behavior.

The animals were randomized and acclimated to housing conditions for 2 weeks prior to the experimental protocols. The animals were fed a standard diet and water ad libitum. All the animals were treated in accordance with the Institute for Laboratory Animal Research Guidelines for the Care and Use of Laboratory Animals. All the experiments were conducted in the Laboratory Animal House, Department of Pharmacology, Faculty of Pharmacy, University of Medicine and Pharmacy at Ho Chi Minh City, Vietnam. This experimental study was approved by the Ethics Committee (decision No. 2441/GCN-HDDDNCTDV) on 20 September 2024.

#### 2.4.2. Acute Toxicity of Vitexin–Isovitexin-Loaded Microspheres

An acute toxicity test was conducted on Swiss albino mice following the OECD 420 guidelines [42]. The mice were randomly divided into 2 groups as follows:

Control group: distilled water, p.o.

mVTX/iVTX 2 group: vitexin–isovitexin-loaded microspheres 2 g/kg p.o. (the highest dose that could pass through the needles).

The mice were fasted overnight and subsequently administered a single dose according to the treatment regimens mentioned above. The animals were observed for 14 days to record mortality rates and any potential signs of toxicity. Body weight was recorded daily.

#### 2.4.3. Hypoglycemic Effects of Vitexin–Isovitexin-Loaded Microspheres

Alloxan monohydrate was freshly prepared before use. The animals were fasted for 12 h prior to injection. The mice were then intravenously (i.v.) injected with alloxan 55 mg/kg (day 2) [43]. The mice were observed for 6 h after injection for any reaction and 0.1 mL an aqueous solution of 5% glucose was administered to those exhibiting signs of tiredness and shivering [44].

Three days post-injection, blood glucose levels were measured (day 1) and mice with blood glucose levels ≥ 200 mg/dL were used for the experiment. The mice were randomly divided into 5 groups, including:

Control group: distilled water, p.o.

ALX group: alloxan (55 mg/kg, i.v.).

GBC group: alloxan (55 mg/kg, i.v.) + glibenclamide (10 mg/kg, p.o.) [45].

mVTX/iVTX 30 group: alloxan (55 mg/kg, i.v.) + vitexin–isovitexin-loaded microspheres (30 mg/kg, p.o.) [46].

mVTX/iVTX 60 group: alloxan (55 mg/kg, i.v.) + vitexin–isovitexin-loaded microspheres (60 mg/kg, p.o.) [46].

Blood glucose levels were measured on days 1, 7, 14, and 21 after hyperglycemia induction. After overnight fasting, blood glucose levels were measured by tail vein blood collection [47], using an ACCU-CHEK Active Glucometer (Hoffmann-La Roche Ltd., Basel, Switzerland).

The area under the curve (AUC) for blood glucose levels on days 1, 7, 14, and 21 was calculated using Formula (3):

(3) $$\mathrm{AUC}\left(\frac{\mathrm{mg}}{\mathrm{dL}}\;\times \;\mathrm{day}\right)=\frac{\mathrm{Sum}\;\mathrm{of}\;\mathrm{blood}\;\mathrm{glucose}\;\mathrm{levels}\;\mathrm{at}\;2\;\mathrm{consecutive}\;\mathrm{time}\;\mathrm{frames}\;\times \;\mathrm{Time}\;\mathrm{interval}}{2}$$

The total AUC of blood glucose levels was calculated as the sum of the incremental AUC [48,49].

On the 21st day of the experiment, the mice were sacrificed and blood samples were collected by cardiac puncture. The blood samples were preserved in EDTA-buffered tubes to prevent coagulation. The pancreas was collected, rinsed with a 0.9% saline solution, and fixed by immersion in 10% buffered formalin.

#### 2.4.4. Oral Glucose Tolerance Test (OGTT)

An oral glucose tolerance test was conducted on normoglycemic Swiss albino mice as previously described [50], with slight modifications. The mice were randomly divided into four groups (n = 8 per group).

Glucose group: glucose (2 g/kg, p.o.).

GBC group: glibenclamide (10 mg/kg, p.o.) [45] + glucose (2 g/kg, p.o.).

mVTX/iVTX 30 group: vitexin–isovitexin-loaded microspheres (30 mg/kg, p.o.) [46] + glucose (2 g/kg, p.o.).

mVTX/iVTX 60 group: vitexin–isovitexin-loaded microspheres (60 mg/kg, p.o.) [46] + glucose (2 g/kg, p.o.).

The mice were fasted for 8 h prior to the experiment, and blood glucose levels were measured at the 0 min time point. Then, apart from the glucose group, the other 3 groups were orally administered the corresponding drugs. After 1 h, all groups received a glucose load of 2 g/kg p.o. Blood glucose levels were assessed at time points of 15, 30, 60, and 120 min after glucose intake.

The area under the curve (AUC) of blood glucose levels at t = 0, 15, 30, 60, and 120 min was calculated using Formula (4):

(4) $$\mathrm{AUC}\left(\frac{\mathrm{mg}}{\mathrm{dL}}\;\times \;\mathrm{minute}\right)=\frac{\mathrm{Sum}\;\mathrm{of}\;\mathrm{blood}\;\mathrm{glucose}\;\mathrm{levels}\;\mathrm{at}\;2\;\mathrm{consecutive}\;\mathrm{time}\;\mathrm{frames}\;\times \;\mathrm{Time}\;\mathrm{interval}}{2}$$

The total AUC of blood glucose levels was calculated as the sum of the incremental AUC [48,49].

#### 2.4.5. Evaluation of the Effects of Vitexin–Isovitexin-Loaded Microspheres on HbA1c, Insulin Concentrations, Insulin Resistance, and β-Cell Function

**HbA1c assay.** The HbA1c assay was performed using the NycoCard^TM^ HbA1c test (1116813, Abbott, Abbott Park, IL, USA) according to the manufacturer’s guidelines.

**Insulin assay.** The insulin assay was performed using an ARCHITECT i1000SR immunoassay analyzer (Abbott, Abbott Park, IL, USA).

**Insulin resistance (IR).** IR was evaluated using the homeostatic model assessment (HOMA-IR) with Formula (5) [48,51]:

(5) $$\mathrm{HOMA-IR}=\frac{\mathrm{Glucose}\;\left(\frac{\mathrm{mg}}{\mathrm{dL}}\right)\times \;\mathrm{Insulin}\;\left(\frac{\mu U}{\mathrm{mL}}\right)}{405}$$

**β-cell function.** β-cell function was evaluated using the homeostatic model assessment (HOMA-β) with Formula (6) [52]:

(6) $$\mathrm{HOMA}\text{-}\beta =\frac{360\;\times \;\mathrm{Insulin}\;(\frac{\mu U}{\mathrm{mL}})}{\mathrm{Glucose}\;\left(\frac{\mathrm{mg}}{\mathrm{dL}}\right)-\;63}$$

#### 2.4.6. Hematoxylin and Eosin (H&E) Staining

Paraffin-embedded pancreas specimens were prepared using a Tissue-Tek^®^ TECTM 5 tissue-embedding module (Sakura Finetek Japan Co., Ltd., Tokyo, Japan) according to a standard protocol. Pancreas specimens were cut into sections of 7.0 μm and stained with hematoxylin and eosin (Sigma-Aldrich, Saint Louis, MO, USA) [43]. Pancreatic histology was evaluated using an ECLIPSE Ci-E microscope (Nikon Instruments Inc., Tokyo, Japan) by an investigator blinded to the experimental groups [43,53]. The image-processing program ImageJ Version 1.54k (NIH Image, https://imagej.net/ij/ (accessed on 19 May 2025)) was used to calculate the areas of Islets of Langerhans. H&E staining was performed at the Cell-Tissue Department, Biomedical Research Center, Pham Ngoc Thach University of Medicine, Ho Chi Minh City.

#### 2.4.7. Statistical Analysis

The results were expressed as mean ± S.E.M. (standard error of mean). Statistical analyses were performed using the unpaired *t*-test for comparisons between two groups and one-way analysis of variance (ANOVA), followed by a Tukey post hoc test for comparisons between more than two groups with GraphPad Prism 9.5 (GraphPad Software Inc., Boston, MA, USA). Differences were considered statistically significant at *p* < 0.05.

## 3. Results

### 3.1. Optimization of Alginate Cores’ Formulation

A total of 36 experiments, designed using response surface methodology and an I-optimal design, were conducted to evaluate the characteristics of the resulting alginate cores, as shown in Table 2.

To evaluate the effect of independent variables on responses, a suitable mathematical model was fitted to investigate the correlated relationships of each response. The analysis mathematic regressions were performed to determine the influence of the variables on the responses and statistically significant models were identified. From Table 3, it can be inferred that the (Y_1_) alginate core size, (Y_2_) loading capacity, (Y_3_) encapsulation efficiency, and (Y_4_) the in vitro release rate after 1 h were fitted for analysis of the influence trends by square root, inverse square root, natural lograrit and power (lambda 3) regression, respectively.

ANOVA analysis was applied to all the responses obtained to study the significance of the model (Supplementary Table S2). The regression coefficient for the suitable model and significance probability (*p*-value) of each factor in the model are given in Table 4.

#### 3.1.1. Effect on Alginate Core Size

**Figure 2:**
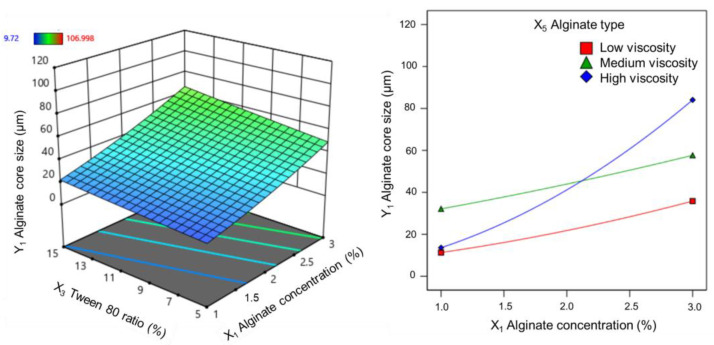
Response surfaces showing the effect of (X_1_) alginate concentration and (X_3_) Tween 80 ratio (**left**), and 2D plots showing the effect of (X_1_) alginate concentration and (X_5_) alginate type (**right**) on the two-factor interaction coefficients of the RSM model for (Y_1_) alginate core size.

The alginate core size is a critical quality factor that influences both the loading and the release capacities of the active ingredient and determines the pharmaceutical feasibility for application in pharmaceutical preparations. The average size of the alginate cores obtained from the 36 experiments ranged from 9.7 µm to 107.0 µm. Correlation analysis revealed that alginate core size was statistically significant for all transformations (*p* < 0.05), with the square root transformation yielding the largest R^2^ coefficient. Simultaneously, the interaction effects were modeled using a two-factor interaction (2FI) regression approach. Among the parameters, alginate concentration had the most significant impact on alginate core size (*p* < 0.0001). The results (shown in Figure 2) demonstrated that alginate concentration and Tween 80 ratio were directly proportional to the average alginate core size. Specifically, when the alginate concentration increased within the range of 1–3%, the alginate core size grew significantly, ranging from 10 to 50 µm.

Additionally, the interaction between alginate concentration and type was also directly proportional to the mean alginate core size. In particular, increasing the concentration of high-viscosity alginate resulted in a more pronounced increase in alginate core size compared to low- and medium-viscosity alginate. The regression coefficients for the variables affecting alginate core size are presented in the following equations, expressed in terms of coded factors:

$$\sqrt{Y_{1}}=5.85\;+1.69X_{1}+0.53X_{3}\;-\;1.25X_{5}\;-\;0.36X_{1}X_{5}\;\;\;\;\;\;\mathrm{(low-viscosity alginate)}$$

$$\sqrt{Y_{1}}=5.85\;+1.69X_{1}+0.53X_{3}+0.73X_{5}\;-\;0.72X_{1}X_{5}\;\mathrm{(medium-viscosity alginate)}$$

$$\sqrt{Y_{1}}=5.85\;+1.69X_{1}+0.53X_{3}+0.52X_{5}+1.08X_{1}X_{5\;}\;\;\;\;\;\;\;\;\mathrm{(high-viscosity alginate)}$$

#### 3.1.2. Effect on Vitexin–Isovitexin Loading Capacity

**Figure 3:**
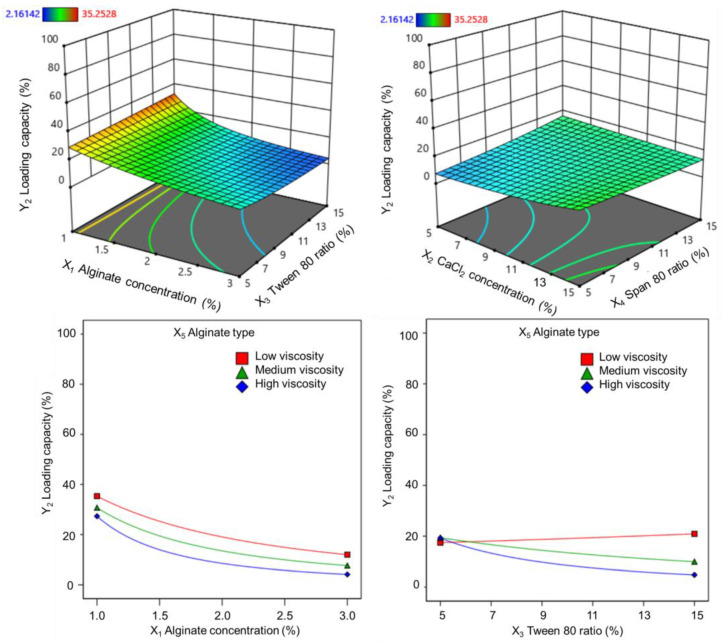
Response surfaces and 2D plots showing the effect of (X_1_) alginate concentration, (X_2_) calcium chloride concentration, (X_3_) Tween 80 ratio, (X_4_) Span 80 ratio, and (X_5_) alginate type on the two-factor interaction coefficients of the RSM model for (Y_2_) loading capacity.

Loading capacity is an important factor that needs to be considered when designing microspheres because it affects the ability to express the therapeutic activity of the dosage form. The amount of vitexin–isovitexin contained in the alginate core obtained from 36 experiments varied greatly, ranging from 2.2% to 35.3%. The loading capacity was significantly correlated with all transforms (*p* < 0.05), with the strongest correlation observed for the inverse square-root transforms (highest R^2^). The ANOVA of the inverse square root transform showed that the loading capacity of the alginate core was affected by the (X_1_) alginate concentration, (X_3_) Tween 80 ratio, and (X_5_) alginate type. In addition, the interactions between the two factors (X_1_.X_3_) alginate concentration and Tween 80 ratio; (X_2_.X_4_) calcium chloride concentration and Tween 80 ratio; (X_3_.X_5_) and Tween 80 ratio and alginate type also significantly affected the vitexin–isovitexin loading capacity of the alginate core (shown in Figure 3). The alginate concentration had the greatest influence on the loading capacity (*p* < 0.0001), whereas the simultaneous influence of calcium chloride concentration and Tween 80 ratio did not significantly change the loading capacity. The interaction between alginate type and Tween 80 ratio was different for each alginate type (shown in Figure 3). For low-viscosity alginate, the loading capacity did not change significantly when the Tween 80 ratio was changed. For medium- and high-viscosity alginates, the loading capacity tended to decrease as the Tween 80 ratio increased from 5% to 15%.

The coefficients of these variables on the loading capacity are given in the regression equations in terms of the coded factors as follows:

$$\frac{1}{\sqrt{Y_{2}}}=0.30+0.10X_{1}+0.05X_{3}\;-\;0.05X_{5}+0.05X_{1}X_{3}+0.04X_{2}X_{4}\;-\;0.06X_{3}X_{5}\;\;\;\;\;\;\;\;\;\mathrm{(low-viscosity alginate)}$$

$$\frac{1}{\sqrt{Y_{2}}}=0.30+0.10X_{1}+0.05X_{3}\;-\;0.01X_{5}+0.05X_{1}X_{3}+0.04X_{2}X_{4}\;-\;0.01X_{3}X_{5}\;\mathrm{(medium-viscosity alginate)}$$

$$\frac{1}{\sqrt{Y_{2}}}=0.30+0.10X_{1}+0.05X_{3}+0.06X_{5}+0.05X_{1}X_{3}+0.04X_{2}X_{4}+0.06X_{3}X_{5}\;\;\;\;\;\;\;\;\;\;\mathrm{(high-viscosity alginate)}$$

#### 3.1.3. Effect on Vitexin–Isovitexin Encapsulation Efficiency

**Figure 4:**
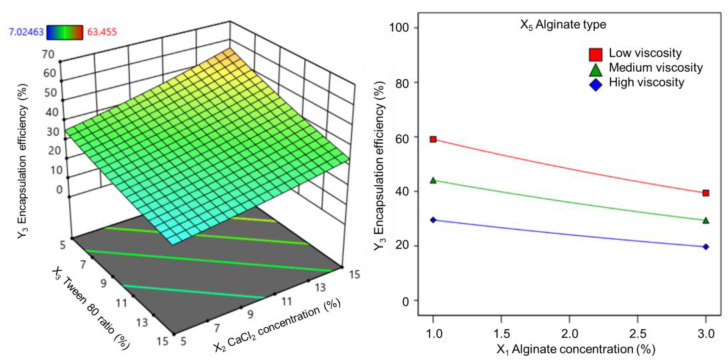
Response surfaces showing the effect of (X_3_) Tween 80 ratio and (X_2_) calcium chloride concentration (**left**), and 2D plots showing the effect of (X_1_) alginate concentration and (X_5_) alginate type (**right**) on the linear coefficients of the RSM model for (Y_3_) encapsulation efficiency.

Similar to the loading ratio, entrapment efficiency is an important parameter for a microsphere system carrying an active ingredient. The entrapment efficiency of vitexin–isovitexin in the alginate cores obtained from 36 experiments ranged from 7.0% to 63.5%. Correlation analysis revealed a statistically significant fit of encapsulation efficiency with all transforms (*p* < 0.05). Among these, the logit transforms exhibited a high correlation coefficient (R^2^), indicating a strong association and suitability for presenting response surfaces with linear regression. The results showed that increasing the Tween 80 ratio in the range of 5–15% decreased the entrapment efficiency, whereas increasing the calcium chloride concentration led to an increase in the entrapment efficiency of the alginate core (shown in Figure 4). In addition, the entrapment efficiency decreased when alginate was used with increasing viscosity, as well as when the alginate concentration was increased in the range of 1–3% (shown in Figure 4).

The coefficients of these variables on encapsulation efficiency are provided in the regression equations in terms of the following coded factors:

$$\ln\left(Y_{3}\right)=3.45\;-\;0.20X_{1}+0.23X_{2}\;-\;0.25X_{3}+0.33X_{5}\;\;\;\;\;\;\;\mathrm{(low-viscosity alginate)}$$

$$\ln\left(Y_{3}\right)=3.45\;-\;0.20X_{1}+0.23X_{2\;}-\;0.25X_{3}+0.04X_{5}\;\mathrm{(medium-viscosity alginate)}$$

$$\ln\left(Y_{3}\right)=3.45\;-\;0.20X_{1}+0.23X_{2}\;-\;0.25X_{3}\;-\;0.36X_{5}\;\;\;\;\;\mathrm{(high-viscosity alginate)}$$

#### 3.1.4. Effect on Vitexin–Isovitexin Release Rate After 1 h

**Figure 5:**
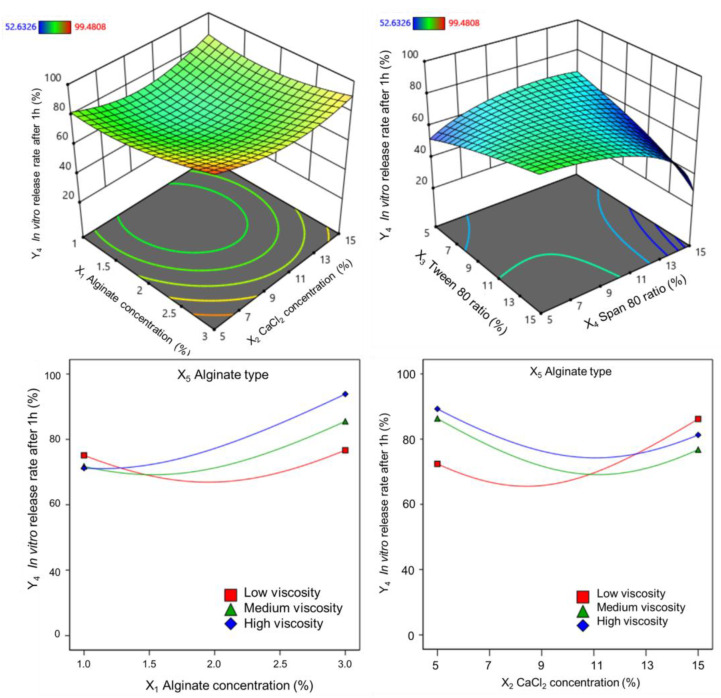
Response surfaces showing the plot of the coefficients on the in vitro release rate after 1 h.

The vitexin–isovitexin release rates of alginate microspheres in 1 h obtained from 36 experiments ranged from 52.6% to 99.5%. Correlation analysis demonstrated a significant correlation between the power (lambda = 3) and the highest correlation coefficient (R^2^). When the alginate concentration was varied in the range of 1–3%, the release rate after 1 h tended to increase slightly (shown in Figure 5).

At the same time, the interaction between alginate concentration and calcium chloride concentration showed a concave curve response surface, and the release rate after 1 h was low when the alginate concentration was low and the calcium chloride concentration was in the range of 9–11%. In addition, the release rate decreased when a low Tween 80 or high Span 80 ratio was used. The higher the viscosity and concentration of the alginate, the higher the release rate. The coefficients of these variables for the release rate after 1 h are given by the following regression equations:

$${\left(Y_{4}\right)}^{3}=3.88\;\times \;10^{5}+1.22\;\times \;10^{5}X_{1}\;-\;1.09\;\times \;10^{5}X_{1}X_{5}+1.45\;\times \;10^{5}X_{2}X_{5}\;-\;1.30\;\times \;10^{5}X_{3}X_{4}+2.01\;\times \;10^{5}{{(X}_{2})}^{2}\;\;\;\;\;\mathrm{(low-viscosity alginate)}$$

$${\left(Y_{4}\right)}^{3}=3.88\;\times {\;10}^{5}+1.22\;\times {\;10}^{5}X_{1}+2771.31X_{1}X_{5\;}-\;76.29\;\times {\;10}^{3}X_{2}X_{5}\;-\;1.30\;\times \;10^{5}X_{3}X_{4}+2.01\;\times \;10^{5}{{(X}_{2})}^{2}\;\mathrm{(medium-viscosity alginate)}$$

$${\left(Y_{4}\right)}^{3}=3.88\;\times {\;10}^{5}+1.22\;\times {\;10}^{5}X_{1}+10.65\;\times {\;10}^{4}X_{1}X_{5\;}-\;68.61\;\times {\;10}^{3}X_{2}X_{5}\;-\;1.30\;\times {\;10}^{5}X_{3}X_{4}+2.01\;\times \;10^{5}{{(X}_{2})}^{2}\;\mathrm{(high-viscosity alginate)}$$

#### 3.1.5. Optimization of the Formulation Parameters

The obtained results and selected mathematical model were used to build the optimization formulation (Table 5). These conditions were repeated three times, and the following characteristics were examined: morphology, alginate core size, loading capacity, encapsulation efficiency, and the in vitro release rate.

The results indicated that the obtained alginate cores from the optimized formulation had characteristics with no significant difference from those obtained with the software suggessions (*p* > 0.05). The optimized alginate cores were then subjected to chitosan encapsulation, resulting in alginate–chitosan microspheres, through a controlled immersion process in a 0.5% chitosan solution (pH 5) for two hours (detailed in the “Materials and Methods” section).

### 3.2. Characterization of the Microspheres

#### 3.2.1. Morphology

**Figure 6:**
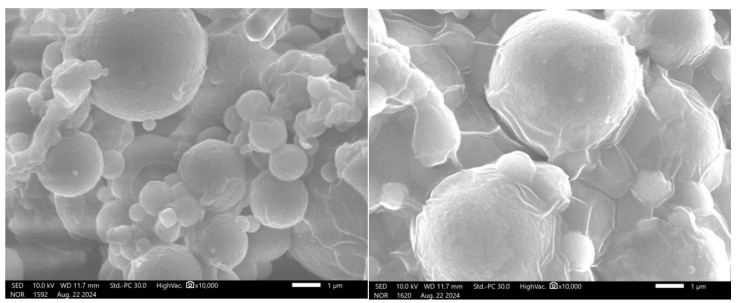
The morphology of alginate cores (**left**) and alginate–chitosan microsphere (**right**) (magnification of 10,000×).

The ion gelation process successfully produced spherical microspheres with micron-sized dimensions. SEM images of the alginate cores revealed smooth, spherical surfaces (shown in Figure 6).

**Figure 7:**
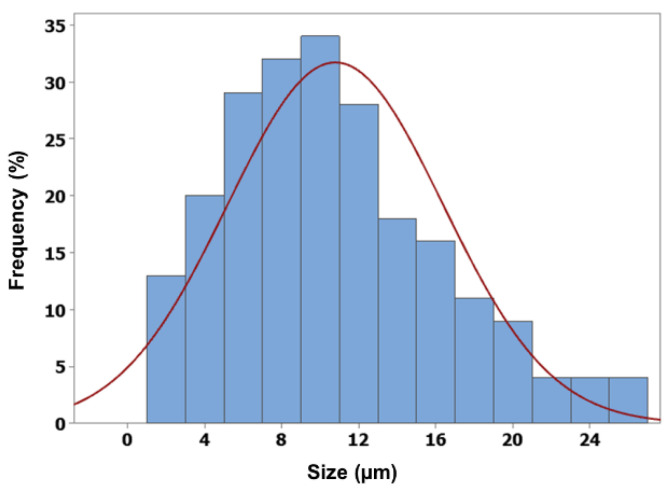
Particle size distribution of alginate–chitosan microspheres.

The morphology of the alginate–chitosan microspheres remained consistent with that of the alginate cores. Additionally, the mean size of the alginate–chitosan microspheres was larger than that of the uncoated cores (10.78 ± 5.59 µm) and exhibited a narrow size distribution (shown in Figure 7). The chitosan coating formed a polyelectrolyte complex, which slowed ion exchange and contributed to a controlled release.

#### 3.2.2. Loading Capacity and Encapsulation Efficiency

The loading capacity and encapsulation efficiency of both microsphere stages were evaluated in a buffer (pH 7.4). The results showed that the loading capacity and encapsulation efficiency of the alginate–chitosan microspheres were 22.45% ± 0.42% and 68.92% ± 1.28%, respectively.

**Figure 8:**
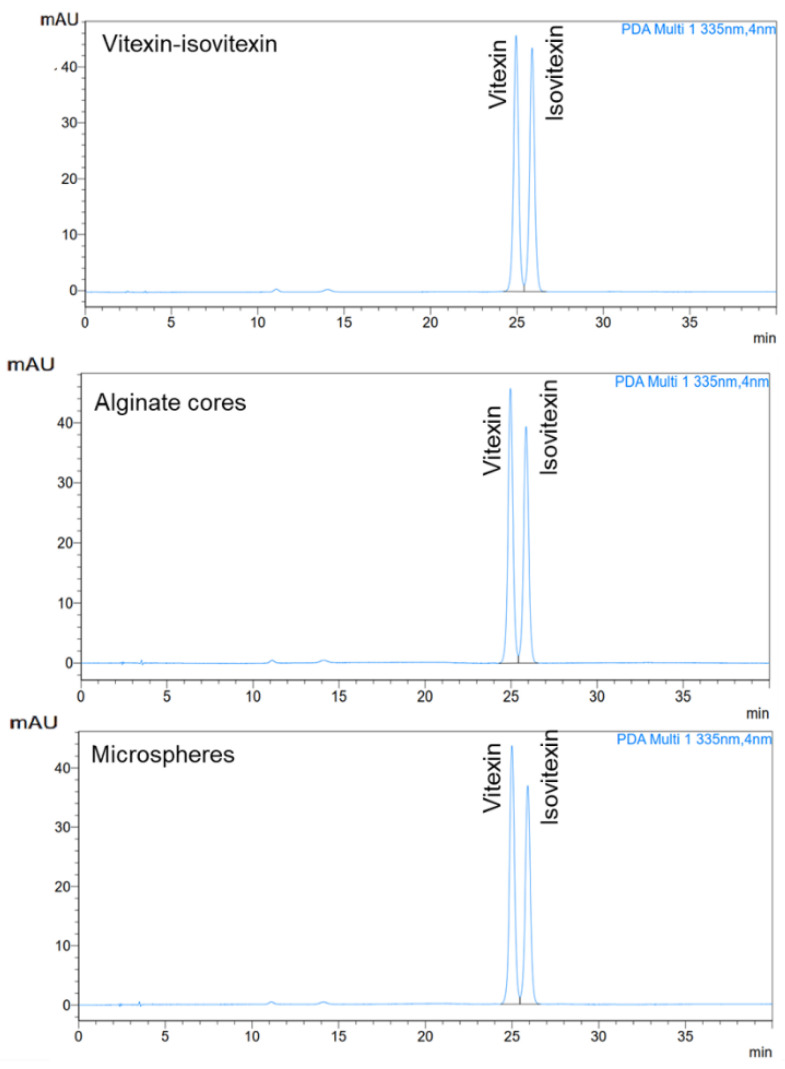
The HPLC chromatography of vitexin–isovitexin (as raw material), alginate cores, alginate–chitosan microspheres.

Furthermore, the HPLC chromatograms (shown in Figure 8) indicated that the initial input ratio of the raw mixture of vitexin and isovitexin during the microsphere preparation was 1:1. However, the analysis of the final microsphere composition revealed a slightly higher proportion of vitexin. Statistical analysis revealed no significant difference between the initial and final vitexin–isovitexin ratios (*p* > 0.05).

#### 3.2.3. In Vitro Vitexin–Isovitexin Release and Kinetics Studies

The in vitro release rates of vitexin and isovitexin from the microspheres were evaluated in a buffer (pH 7.4) over 8 h. Additionally, a two-step process simulating the digestive environment (pH 1.2 and pH 6.8), as outlined in the US Pharmacopoeia (USP 43, general monograph <711> Dissolution), was performed to compare the release profiles of the alginate–chitosan microspheres with the optimized alginate cores.

**Figure 9:**
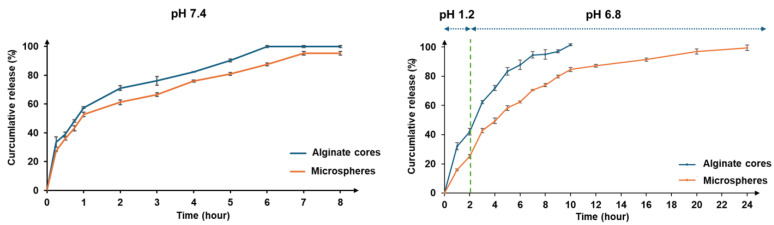
The in vitro release profiles of the alginate cores, alginate–chitosan microspheres in different pH buffers. Data were presented as mean ± SD (n = 3).

The results (shown in Figure 9) showed that the controlled release of vitexin–isovitexin from the microspheres was sustained up to 8 h in pH 7.4 (approximately 95.19%) and extended to 24 h in the digestive medium (pH 1.2 and 6.8), achieving 99.53%. In comparison, the optimized alginate cores exhibited a rapid release profile. The alginate–chitosan microspheres demonstrated a more controlled release profile, with approximately 25.1% release at pH 1.2 and 99.5% release at pH 6.8.

**Figure 10:**
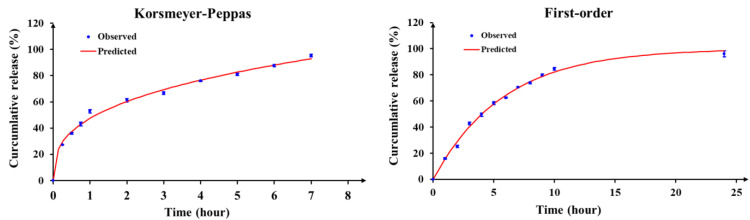
The in vitro release profile of microspheres following the kinetic models. Data were presented as mean ± SD (n = 3).

The in vitro release data of alginate–chitosan microspheres were analyzed according to kinetic models to find the best fit model for the control release (Table 6). The results revealed that the release of microspheres in pH 7.4 was consistent with the Korsmeyer–Peppas model. The release of vitexin–isovitexin was by the diffusion mechanism according to Fick’s law. Meanwhile, the in vitro release of microspheres in the digestive medium (pH 1.2 and 6.8) was fit to the first-order kinetic model and released vitexin–isovitexin via the diffusion mechanism according to Fick’s law (shown in Figure 10).

#### 3.2.4. Zeta Potential

**Figure 11:**
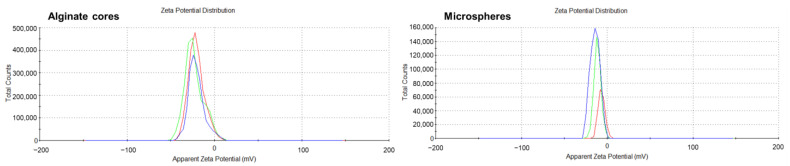
The zeta potential of alginate cores and alginate–chitosan microspheres (n = 3).

The zeta potential of the alginate–chitosan microspheres was determined from the surface charge, as shown in Figure 11. There was a significant change in the surface charge of the alginate–chitosan microspheres (−6.93 ± 0.25 mV, n = 3) compared to the highly negative charge on optimized alginate cores (−20.7 ± 2.60 mV, n = 3), demonstrating that there were interactions between alginate and chitosan. The carboxylic groups of alginate on the microsphere surface were neutralized upon interaction with the amino groups of chitosan. While not entirely neutral, the surface charge of the microspheres became significantly less negative, indicating a successful coating and a shift towards neutrality.

#### 3.2.5. DSC

**Figure 12:**
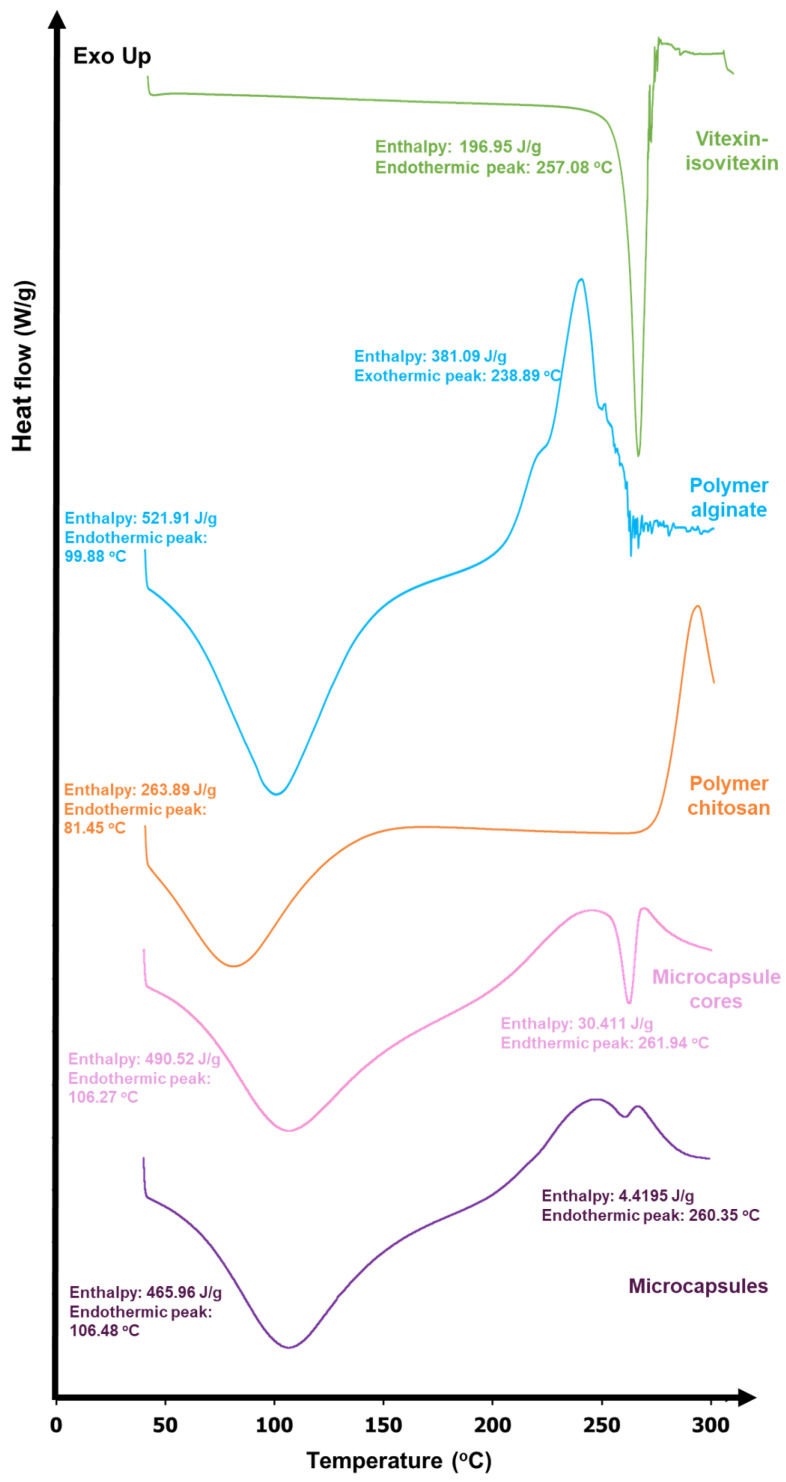
DSC thermogram of vitexin–isovitexin (as raw material), alginate, chitosan, alginate cores, and alginate–chitosan microspheres.

The DSC thermograms of vitexin–isovitexin, alginate, chitosan, alginate cores, and alginate–chitosan microspheres are shown in Figure 12. The DSC curve of vitexin–isovitexin displayed an endothermic peak at approximately 257.08 °C, with an enthalpy of 196.95 J/g. The DSC analysis of the alginate polymer showed a broad endothermic peak at 100 °C, attributed to the evaporation of residual water, with a required enthalpy of 521.91 J/g, and an exothermic peak at 239 °C, indicating crystallization, with an enthalpy of 381.09 J/g.

Similarly, the DSC of chitosan polymer exhibited a broad endothermic peak at 81 °C, related to the evaporation of residual water or solvent, with an enthalpy of 263.89 J/g. An exothermic peak, attributed to recrystallization, was observed near 300 °C. The DSC thermogram of the alginate cores showed two distinct endothermic peaks at approximately 106 and 262 °C, with enthalpies of 490.52 and 30.41 J/g, respectively. The DSC thermogram of the alginate–chitosan microspheres was similar, displaying two endothermic peaks at approximately 106 and 260 °C. However, the second endothermic peak required a smaller enthalpy (4.42 J/g) compared to that of the alginate cores.

### 3.3. Acute Toxicity and Hypoglycemic Effects of Vitexin–Isovitexin-Loaded Microspheres

#### 3.3.1. Acute Toxicity of Vitexin–Isovitexin-Loaded Microspheres

**Figure 13:**
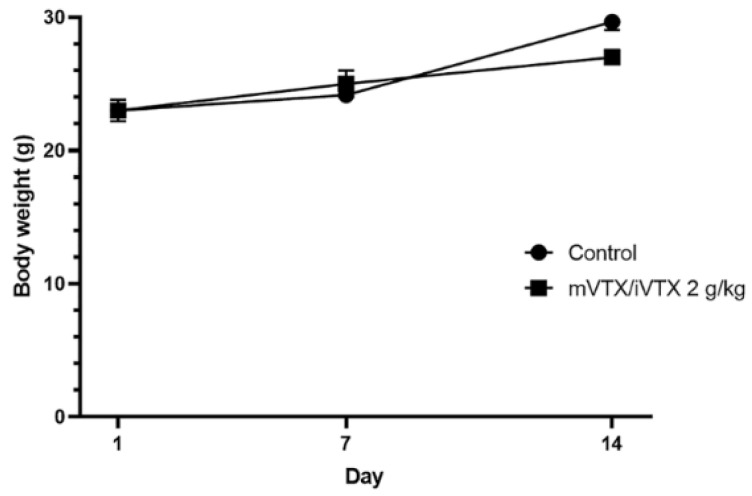
The body weight of mice in 14 days. Data were presented as mean ± S.E.M. (n = 6). Comparisons between the groups were carried out by an unpaired *t*-test. mVTX/iVTX = vitexin–isovitexin-loaded microspheres.

Following the OECD guideline, three doses of vitexin–isovitexin (2 g/kg, 5 g/kg, 10 g/kg) (Supplementary Figure S3) and its microsphere form (maximal dose through needles) (shown in Figure 13) were evaluated for any signs of acute toxicity. During the 14-day observation, mice treated with vitexin–isovitexin-loaded microspheres 2 g/kg showed no mortalities and no significant changes in appearance, behavioral patterns, water and food consumption when compared with the mice in the control group. In terms of body weight, the mVTX/iVTX 2 g/kg group had slightly lower body weights than their control counterpart at the 14th day; however, no statistical significance was recorded. As the limit dose 2 g/kg for the microsphere form showed safety on mice, their LD_50_ values for oral toxicity could not be determined.

#### 3.3.2. Effect of Vitexin–Isovitexin-Loaded Microspheres on Blood Glucose Levels

According to Table 7, apart from the control group, all alloxan-injected groups had their blood glucose levels elevated above 200 mg/dL on day 1 of the experiment. The blood glucose level of the ALX group tended to increase throughout the experiment and reached a peak of above 400 mg/dL after 21 days. The mVTX/iVTX 30 and 60 groups exerted a lowering effect on blood glucose levels on day 21. Mice treated with microspheres at these two doses showed a remarkable decline in blood glucose levels when compared to the ALX group (*p* = 0.0046 and *p* < 0.0001 vs. ALX group, respectively). In addition, the 60 mg/kg dosage appeared to be more effective than the 30 mg/kg group, though no statistically significant difference was observed between the two treatment groups.

Table 8 demonstrates the AUC of blood glucose levels in mice from day 1 to day 21. Compared to the ALX group, the 3-week treatment with glibenclamide and vitexin–isovitexin-loaded microspheres at two doses (30 and 60 mg/kg) remarkably decreased the AUC of blood glucose levels compared to the untreated group (glibenclamide: *p* = 0.0031 vs. ALX; microspheres 30 mg/kg: *p* = 0.0393 vs. ALX; and microspheres 60 mg/kg: *p* < 0.0001 vs. ALX, respectively). These results indicate that the formulated microspheres, particularly at the higher dose, exhibited a substantial and statistically significant reduction in overall blood glucose exposure over the 21-day treatment period, comparable to the effect of the standard drug, glibenclamide, when compared to the untreated diabetic control group (ALX).

#### 3.3.3. Effect of Vitexin–Isovitexin-Loaded Microspheres on Oral Glucose Tolerance

**Figure 14:**
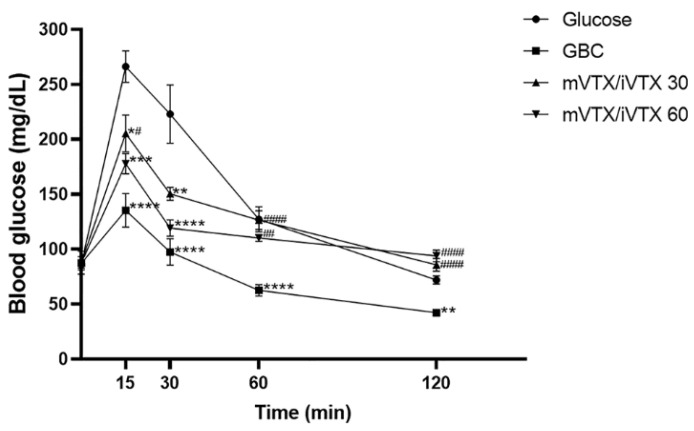
Effect of vitexin–isovitexin-loaded microspheres on oral glucose tolerance in alloxan-induced hyperglycemic mice. Data were presented as mean ± S.E.M. (n = 8). Comparisons between the groups were carried out by a one-way analysis of variance (ANOVA), followed by a Tukey post hoc test. * *p* < 0.05, ** *p* < 0.01, *** *p* < 0.001, **** *p* < 0.0001 vs. glucose group. ^#^ *p* < 0.05, ^##^ *p* < 0.01, ^####^ *p* < 0.0001 vs. GBC group. GBC = glibenclamide, and mVTX/iVTX = vitexin–isovitexin-loaded microspheres.

Figure 14 demonstrates the changes in blood glucose levels of four groups of mice after 0, 15, 30, 60, 120 min since glucose intake. It can be seen that all groups showed an increase in blood glucose levels after 15 min. The blood glucose levels started to decrease after 30 min, with the levels of three treatment groups being considerably lower than those of the glucose group (*p* < 0.01). This downward trend in blood glucose levels continued until the 120 min point, when the values of all groups were roughly equivalent to 0 min point, except for the GBC group. The GBC group experienced a sharp decline in the blood glucose level after 120 min.

The AUC of blood glucose levels in the oral glucose tolerance test (OGTT) was also calculated and shown in Table 9. All the treatment groups showed a decrease in the AUC when compared to the glucose group in the 120 min period, among which only administration with glibenclamide and microspheres 60 mg/kg brought about statistically significant differences (*p* < 0.0001 and *p* = 0.0238 vs. glucose group, respectively).

#### 3.3.4. Effect of Vitexin–Isovitexin-Loaded Microspheres on HbA1c and Insulin Levels

According to Table 10, the HbA1c levels of the ALX group and all treatment groups were considerably higher than those of the control group (*p* < 0.0001). It could be seen that the treatment groups demonstrated an ameliorative effect on the HbA1c level, as the values of these three groups were slightly lower than those of the untreated group. However, no statistically significant difference was observed between the treatment groups.

There was no significant difference in insulin levels between groups. However, the untreated group still showed a slight decrease in insulin levels compared with the control group. In contrast, the treatment groups displayed a rising tendency in insulin levels compared to the ALX group, although the differences were not statistically significant. The increase in insulin levels, although not remarkable, corresponded with a decline in blood glucose levels (Table 8), as described above.

#### 3.3.5. Effect of Vitexin–Isovitexin-Loaded Microspheres on Insulin Resistance and β-Cell Function

According to Table 11, the ALX group had a considerably higher HOMA-IR index than that of the control group (*p* = 0.0038 vs. control group). All the treatment groups using glibenclamide and vitexin–isovitexin-loaded microspheres showed a decreasing tendency in HOMA-IR in comparison with the untreated group. However, there was no statistically significant difference between the treatment groups and the ALX group. In terms of the HOMA-β index, the ALX group showed a decline in this figure when compared to the control group. In contrast, the treatment groups displayed a rising tendency in HOMA-β in comparison with the untreated group. However, no statistically significant differences were observed between the groups.

#### 3.3.6. Effect of Vitexin–Isovitexin-Loaded Microspheres on Islets of Langerhans

**Figure 15:**
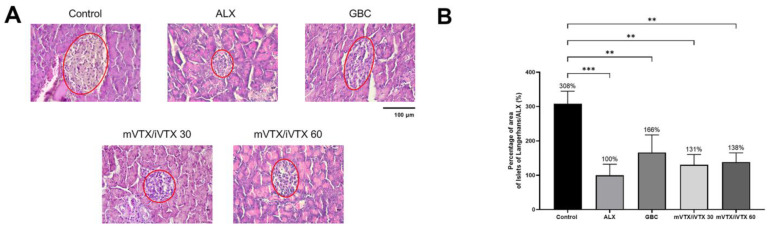
Effect of vitexin–isovitexin-loaded microspheres on the Islets of Langerhans. (**A**). Histomicrographs of pancreases at 40× magnification. (**B**). Percentage of area of Islets of Langerhans in other groups compared to the ALX group (n = 3). ALX = alloxan, GBC = glibenclamide, and mVTX/iVTX = vitexin–isovitexin-loaded microspheres. Comparisons between the groups were carried out by a one-way analysis of variance (ANOVA), followed by a Tukey post hoc test. ** *p* < 0.01, *** *p* < 0.001 vs. control group. Red oval = Islets of Langerhans. Scale bar = 100 μm.

Figure 15A demonstrates the size of the pancreatic Islets of Langerhans in the five groups, while Figure 15B illustrates the percentage of the islet area relative to the ALX group. The control group exhibited normal islet architecture, whereas the hyperglycemic untreated group displayed a diminished islet in terms of size. Treatment groups showed an increase in islet area compared to the ALX group, though the differences were not statistically significant. The dose of 60 mg/kg of vitexin–isovitexin-loaded microspheres appeared to be slightly more effective than the 30 mg/kg one in enhancing islet size, as the percentages of area of islets vs. the ALX group were 138% and 131%, respectively (Figure 15B).

## 4. Discussion

This study focused on developing an alginate–chitosan microsphere drug delivery system. Both polymers exhibit biocompatibility, low toxicity, and have been extensively investigated for their influence on microsphere structure, making them suitable for pharmaceutical applications. A two-stage alginate–chitosan microencapsulation technique was employed to precisely control the morphology and size of the microspheres through a combination of emulsion and ion gelation processes. Subsequently, a coating process was applied to optimize the release kinetics of the encapsulated active ingredients. The active ingredients selected for this study were vitexin and isovitexin, poorly water-soluble flavonoids extracted and purified from medicinal herbs. Despite the recognized pharmacological potential of these compounds, limited research has been conducted on their encapsulation within alginate–chitosan microsphere systems. The optimization of the alginate–chitosan microsphere formulation presented in this study is distinct from previously published research, offering a unique contribution to the field of drug delivery.

### 4.1. Optimization of Alginate Cores’ Formulation

The preparation process for the alginate cores was inspired by the work of Meixia Jin et al. [54] and Design Expert v.13.0.5.0 software was utilized. Design of Experiment (DoE) methodologies were employed to ensure a systematic and efficient approach to the experimental design and analysis, maximizing this study’s impact [55]. An I-optimal response surface design was selected due to its suitability for models incorporating both quantitative and qualitative factors, as well as for scenarios involving varying levels of independent variables. This design prioritizes predictive accuracy, offering advantages over other algorithms such as D-optimal or G-optimal [56,57]. A quadratic regression model was employed as the highest-order model to comprehensively assess the interactions between independent variables and their impact on response variables.

As demonstrated by Alshora et al. [58], particle size played a crucial role in enhancing the solubility of active ingredients by increasing their surface area. In the context of alginate microspheres, particle size significantly influenced both drug loading capacity and release rate [59]. Moreover, a uniform particle size distribution reflected the consistency and efficiency of the microencapsulation process. Alginate concentration was widely recognized as a primary factor affecting the physicochemical properties of alginate microspheres, including morphology, size, and encapsulation efficiency [60]. The findings of this study aligned with this established knowledge, confirming the influence of both alginate concentration and type on alginate core size. Previous studies have reported similar trends. Mokhtari et al. [61] investigated the impact of alginate concentration on the properties of nanoparticles prepared by the internal gelation emulsion method, demonstrating a significant boost in particle size from 512 to 4303 nm as the alginate concentration was increased from 0.5 to 1.0% (*w*/*v*). Similarly, Shukla et al. [62] also reported a proportional relationship between alginate concentration (3 to 5%, *w*/*v*) and microsphere size (417.8 to 453.9 µm) produced by the ionized emulsion method. These findings suggested that alginate polymer concentration exerted a substantial influence on the size and distribution polydispersity index of nanoparticles formed through emulsion techniques. This effect can be attributed to the interaction between the carboxylate functional groups (COO^−^) of the alginate chains and calcium ions. Higher alginate concentrations lead to an increased number of COO^−^ groups and the formation of multiple alginate layers around calcium cations, resulting in the formation of larger particles. Higher alginate concentrations also resulted in increased solution viscosity and decreased shear stress, leading to larger emulsion droplets. This explained the influence of alginate viscosity on alginate core size. Additionally, the interaction between alginate type and concentration significantly impacted core size. High-viscosity alginates exhibited a more pronounced increase in core size with increasing concentration compared to that of low- and medium-viscosity alginates.

Alginate concentration significantly impacts the quality indicators of alginate cores, particularly the loading capacity and encapsulation efficiency of the active substance. In this study, increasing alginate concentration primarily reduced the loading ratio without significantly affecting encapsulation efficiency. This finding aligns with previous research by Kalalo et al. [63], which demonstrated that higher alginate concentrations increase the solution’s viscosity, leading to thicker emulsion droplets and reduced quercetin loading. Similarly, Silva et al. [64] observed no significant change in insulin encapsulation efficiency with increasing alginate concentration. Essifi et al. [65] further elucidated this phenomenon. They reported that higher alginate concentrations increase encapsulation efficiency while reducing loading capacity. This is attributed to the formation of denser microsphere structures with more cross-links between calcium ions and alginate chains. Additionally, the interaction between alginate concentration and Tween 80 ratio influences vitexin–isovitexin loading. Independently, increasing Tween 80 tends to slightly decrease loading. However, at low alginate concentrations, increasing Tween 80 can paradoxically increase active ingredient loading.

Alginate type significantly influenced both the loading capacity and encapsulation efficiency. Low-viscosity alginate yielded alginate cores with higher loading and entrapment than medium- and high-viscosity alginates. Combining low-viscosity alginate with a lower concentration further optimized these parameters. Additionally, the interaction between Tween 80 ratio and alginate type was significant. Although low-viscosity alginate was minimally affected by the Tween 80 ratio, medium- and high-viscosity alginates exhibited decreased loadings with increasing Tween 80 content. This is attributed to the increased polymer chain self-interaction, reduced available space for active ingredient incorporation, and potential loss due to decreased particle surface tension [66].

Calcium chloride, a critical cross-linking agent in alginate-based emulsion gelation, forms a stable three-dimensional “egg-box” network [67]. While the calcium chloride concentration did not influence the loading rate in this study, it exhibited a positive correlation with the encapsulation efficiency. This aligns with previous studies by Mokhtari et al. [61] and Hu et al. [68], who demonstrated an increased encapsulation efficiency of phenolic extracts and curcumin, respectively, at higher calcium chloride concentrations. This effect is attributed to the formation of a dense network of cross-links on the microsphere’s surface.

Alginate concentration significantly influenced the initial in vitro release rate. Increasing alginate concentration from 1% to 2% slightly decreased the in vitro release rate, while further increasing to 3% led to an increased the in vitro release rate. This trend is consistent with previous findings [69], which suggest that higher alginate concentrations can lead to larger alginate cores with more active molecules closer to the surface, resulting in a rapid initial release. Similarly, increasing calcium chloride concentration can also impact the in vitro release rate. This is likely due to the formation of a more porous particle structure, facilitating faster release (brust release). The alginate type, which influences solution viscosity, also affects the in vitro release rates. Higher-viscosity alginate resulted in a higher in vitro release rate. This is consistent with the general understanding that ion-gelated alginate particles often exhibit rapid initial release due to their porous structure [70].

Tween 80 and Span 80 were selected as the emulsifiers to stabilize the emulsion and reduce the interfacial tension between the oil and water phases. Tween 80 facilitates calcium-ion diffusion into the dispersed phase, promoting cross-linking and microsphere solidification. Span 80 primarily stabilizes emulsion droplets by adsorbing them onto their surfaces, preventing agglomeration and enhancing the alginate core structure. Among the formulation parameters, alginate concentration, Tween 80 ratio, and alginate type had the most significant effects on microsphere quality. In contrast, the calcium chloride concentration and Span 80 ratio had less-pronounced effects on the final alginate core properties.

### 4.2. Characterization of the Microspheres

The ion gelation process successfully produced spherical microcapsules with micron-sized dimensions. Alginate–chitosan microcapsules exhibited controlled release of vitexin–isovitexin in pH 7.4, following a diffusion-controlled mechanism, as described by the Korsmeyer–Peppas model. The chitosan coating formed a polyelectrolyte complex, slowing down ion exchange and contributing to the controlled release. Additionally, the higher solubility of vitexin–isovitexin in alkaline pH accelerated the release. Two-stage release experiments simulating digestive pH further highlighted the effectiveness of the polyelectrolyte complex. The microcapsule core exhibited rapid release, while the alginate–chitosan microcapsule demonstrated a more controlled release profile, with approximately 25% release at pH 1.2 and 96% release at pH 6.8. This behavior aligns with first-order kinetics and a diffusion-controlled mechanism. These findings are consistent with previous studies on alginate–chitosan microspheres loaded with various active ingredients, such as lidocaine [71], galactagogue extract [72], nifedipine [73], and cannabis extract [74]. These studies also reported controlled-release profiles and mechanisms similar to those observed in the current research. The prolonged release of the active ingredient from the microspheres was attributed to changes in the properties of both alginate and chitosan polymers. While a simple mechanical coating of chitosan might have led to rapid dissolution at pH 1.2, the formation of a polyelectrolyte complex between alginate and chitosan resulted in a more controlled release profile. Sæther et al. [75] described the mechanism of this composite film formation, which involves dipole interactions and hydrogen bonding between the carboxyl groups of alginate and the amine groups of chitosan. This complexation reduces the porosity of alginate and enhances its mechanical strength, leading to a controlled release of active ingredients in a dynamic pH environment.

The negative zeta potential of the alginate cores, as observed in this study, is consistent with the properties of alginate polymers. This negative charge is attributed to the presence of carboxyl groups in the alginate structure, which ionize to form negatively charged carboxylate groups. This negative charge contributes to the stability of the microspheres in solution by repelling each other and preventing aggregation. Previous studies have also reported negative zeta potentials for alginate-based nanoparticles. For example, Zimet et al. [76] observed zeta potentials in the range of −38.7 to −33.22 mV for Nisaplin-containing alginate nanoparticles, while Bhunchu et al. [77] reported values between −27.8 and −19.8 mV for alginate nanoparticles. These findings further corroborate the negative surface charge of alginate-based particles. The significant decrease in zeta potential from −20.7 mV to −6.93 mV upon chitosan coating indicates a substantial reduction in the negative surface charge of the microspheres. This reduction is attributed to the interaction between the negatively charged carboxyl groups (COO^−^) of alginate and the positively charged amino groups (NH_3_^+^) of chitosan. This interaction results in the formation of a polyelectrolyte complex, which effectively neutralizes the negative charge on the microsphere surface. This complexation has implications for the stability, release properties, and biocompatibility of the microspheres.

Thermal analysis revealed that the first endothermic peak at 106 °C corresponded to the loss of water from the alginate polymer, while the second peak at 262 °C represented a combination of the melting peak of vitexin–isovitexin (257 °C) and the crystallization peak of alginate (239 °C). The upward trend in the baseline further confirmed the presence of alginate in the core. Similarly, the alginate–chitosan microspheres exhibited two endothermic peaks. The first peak corresponded to the melting of both alginate and chitosan polymers, while the second peak required less energy compared to the alginate cores, likely due to the presence of chitosan. Chitosan, with a crystallization onset at 276 °C, reduced the overall energy absorption of the microspheres. Thermal analysis confirmed the presence of alginate, chitosan, and vitexin–isovitexin in the final microsphere structure.

Within the scope of the current study, the effects of chitosan concentration and the resulting outer layer thickness on the release behavior and zeta potential of the microspheres were not explicitly investigated. However, it is acknowledged that these factors are crucial for a more comprehensive understanding of the microsphere system and are essential for further optimizing the coating process in future research. Additionally, stability evaluation is considered a critical step for the future development and potential clinical translation of this formulation. The stability tests, including assessment under various environmental conditions (e.g., temperature, humidity) and over extended periods, are intended to be performed in subsequent research. This will provide crucial evidence regarding the shelf-life and quality attributes of the microspheres over time.

### 4.3. Acute Toxicity and Hypoglycemic Effects of Vitexin–Isovitexin-Loaded Microspheres

This result resonated with the study of Choo et al. [10], which reported that vitexin and isovitexin at the dose of 2 g/kg showed safety on both normoglycemic and induced diabetic rats. According to the Globally Harmonized System (GHS) of Classification and Labelling of Chemicals, isovitexin and vitexin can be classified into Category 5, which is considered relatively safe following acute exposure. In addition, to the best of our knowledge, this is the first study on the safety profile of vitexin and isovitexin in the microsphere form.

Vitexin and isovitexin have long been proven to possess hypoglycemic effects through several mechanisms, including antioxidant activity as flavones [8], α-glucosidase [10], and aldose reductase [13,14,15] inhibition. Several studies have been conducted, both in vitro and in vivo, to confirm the antidiabetic capacity of these two compounds [2,10,11,16]. Despite their enormous potential as antidiabetic agents, vitexin and isovitexin have been investigated separately, and there has not been any research carried out on their combination, let alone on the microsphere form. Therefore, it is important to mention that this is the first study ever to evaluate the bioactivity of vitexin and isovitexin as a combination, especially the hypoglycemic effect.

Mice receiving vitexin–isovitexin microspheres at both doses (30 and 60 mg/kg) showed a clear decrease in blood glucose levels and AUC after 21 days. The hypoglycemic effects of this preparation may be attributed to the pharmacological activities of vitexin and isovitexin themselves [2], combining with the bioavailability-enhancing actions of the microspheres. This result is consistent with those of various studies that support the antidiabetic effects of vitexin and isovitexin [2,10,11,16]. In addition, the blood-glucose-modulating activity of the microsphere form appeared to be dose-dependent, as a higher dose of 60 mg/kg resulted in a greater improvement in blood glucose than the 30 mg/kg dose. Our findings are consistent with those of other studies, which reported that vitexin and isovitexin reduced postprandial blood glucose levels in a dose-dependent manner [10,16].

Glucose tolerance tests have long been important tools for identifying impairments in glucose homeostasis [78,79]. Moreover, as the need for novel antidiabetic medications has become increasingly strong, glucose tolerance tests, especially oral administration, are being utilized to study the effectiveness of new treatments for elevated blood glucose levels [80]. OGTT evaluates the glucose uptake in peripheral tissues and a rise in glucose concentration during OGTT implies a reduction in insulin response as well as in the insulin-secreting ability of pancreatic β-cells [81]. Our results demonstrate that the microsphere form of the vitexin–isovitexin combination alleviated glucose intolerance. These findings are in agreement with those of another study by Choo et al. [10], who also examined the influence of vitexin and isovitexin as separate compounds on glucose tolerance. However, the glucose tolerance test in the study by Choo et al. was performed on both normoglycemic and diabetic rodents, whereas our study only investigated non-diabetic mice. Therefore, the effects of vitexin–isovitexin-loaded microspheres on glucose tolerance should also be tested in animal models of diabetes.

HbA1c or glycated hemoglobin is a biochemical index used to monitor blood glucose levels [81], and HbA1c tests are commonly performed at 3-month intervals in humans. In mouse models, HbA1c concentration is mostly evaluated after an experimental period of over 40 days [43,82,83]. However, in the alloxan-induced hyperglycemia model, HbA1c levels should be measured at an earlier time point of 14–21 days considering the high mortality rate of this model [43]. In this study, mice treated with vitexin–isovitexin-loaded microspheres showed a decrease in HbA1c levels compared with those of untreated mice. Vitexin has been shown to reduce HbA1c levels, as stated in a study by Gayathri et al. [84], which was conducted on high-fat diet-streptozotocin-induced diabetic rats. Regarding isovitexin or the combination of these two compounds, the determination of their effects on HbA1c remains limited. In this study, the state of hyperglycemia in the ALX group resulted from the damage of alloxan to the β-cells, which led to the reduction in insulin secretion. Vitexin has been proven to improve insulin signaling, which could be due to the recovery of islet size (Figure 15) [85]. This might explain the rise in insulin levels in microsphere-treated groups compared to the ALX group, though not remarkable.

HOMA-IR and HOMA-β indices are widely used tools for assessing insulin sensitivity and pancreatic β-cell function, as they are calculated from fasting glucose and insulin levels [51,86,87]. Alloxan, a commonly used diabetogenic agent, induces hyperglycemia through two primary mechanisms. First, it acts as a glucose analog, selectively accumulating in β-cells through the GLUT2 glucose transporter, where it provokes reactive oxygen species (ROS) formation, leading to β-cell deterioration. Second, as a thiol reagent, alloxan inhibits glucose-induced insulin production by targeting the enzyme glucokinase in β-cells [88]. This β-cell damage restricts insulin secretion, resulting in a decreased HOMA-β value. Our findings align with these mechanisms, as the HOMA-β index in the ALX group was lower than in the control group (Table 11). The moderate increase in HOMA-β observed in the treatment groups may reflect partial regeneration of β-cells, which also corresponds with the slight rise in insulin levels noted earlier (Table 11). Various in vitro studies have demonstrated that vitexin protects pancreatic β-cells through mechanisms such as improving insulin signaling [85] and reducing ROS and lipid peroxidation [89]. Regarding the HOMA-IR index, the administration of alloxan reduced insulin sensitivity, leading to an increase in this score (Table 11), as confirmed by previous studies [39,43]. In contrast, treatment with vitexin–isovitexin-loaded microspheres resulted in a slight decrease in HOMA-IR, indicating their capacity to enhance insulin sensitivity and promote peripheral glucose utilization. This impact on HOMA-IR may contribute to the hypoglycemic mechanisms of vitexin–isovitexin-loaded microspheres alongside β-cell-stimulating [85] and α-glucosidase inhibitory (Supplementary Figure S1) effects of vitexin/isovitexin.

However, the lack of statistically significant differences in HbA1c, insulin levels, HOMA-IR, and HOMA-β between the treated groups and the ALX group might be due to the duration of this study. According to several studies, the alloxan model is conducted in less than a month [90,91] due to high rate of mortality [38,43]. This short period of time may lead to the statistical insignificance in some critical parameters, such as HbA1c, insulin, HOMA-IR and HOMA-β. The high mortality rate resulted in the loss of animals in each group, which also caused the lack of statistically significant differences in these indices. Another drawback of the alloxan model relates to its instability, as the half-life of alloxan is short [88]. Due to the limitation of the alloxan model, further investigation on other models of type 2 diabetes needs to be emphasized. The combination of the streptozotocin model and a high-fat diet is usually utilized to simulate type 2 diabetes. First, this model might last for 3 months, which is an appropriate timespan to evaluate the changes in HbA1c, insulin level, HOMA-IR and HOMA-β. These parameters were recorded to be significantly different after at least 40 days [83]. Second, the mortality rate of the streptozotocin–high-fat diet model is lower than that of the alloxan model [92]. This characteristic might contribute to the significance of data in this model through maintaining the number of animals. Third, the chemical stability, diabetogenic index and reproducibility of streptozotocin were proved to be more stable than those of alloxan. Future studies should investigate the effects of the microspheres over longer treatment durations, with varying doses, increase the size of samples, and use different animal models to better understand their impact on insulin dynamics and long-term glycemic control.

Vitexin has been proven to exert protective effect on pancreatic β-cells [85], which might lead to islet size enhancement. Moreover, the H&E staining results are in line with the research of Wang et al. [93], which showed the effects of vitexin on improving deteriorated islets. Similar findings have been reported, with vitexin promoting islet regeneration in streptozotocin-induced diabetic models [94].

To the best of our knowledge, this study represents the first successful optimization of alginate–chitosan microspheres containing a mixture of vitexin and isovitexin, systematically evaluated through their physicochemical characteristics. Furthermore, this is the first study that evaluates the hypoglycemic effects of vitexin and isovitexin combination in microspheres via an improvement in islet size, insulin sensitivity and resistance. The underlying mechanisms might be related to the increase in oral bioavailability of this specific encapsulated form [95].

## 5. Conclusions

Our research successfully developed alginate–chitosan microspheres containing vitexin and isovitexin to control the release and maintain the pharmacological efficacy of these compounds. The microspheres had a spherical mean size of 10.78 µm, with vitexin–isovitexin loading rates of 22.45% and an encapsulation efficiency of 68.92%. In vitro studies demonstrated controlled release for 8 h at pH 7.4 and up to 24 h under simulated gastrointestinal conditions. In vivo, the microspheres exhibited no acute toxicity in Swiss albino mice and demonstrated significant hypoglycemic effects over 21 days at doses equivalent to 30 and 60 mg/kg of vitexin–isovitexin. Furthermore, these doses improved the size of Islets of Langerhans, enhanced pancreatic β-cell function, and reduced insulin resistance. In conclusion, vitexin–isovitexin-loaded microspheres show great promise as a novel treatment for diabetes, offering enhanced oral bioavailability and improved glycemic control.

## Figures and Tables

**Table 1:** Coded units for I-optimal design.

Factor	Unit	Level		
		Lower	Medium	Upper
(X_1_) Alginate concentration	% (*w*/*w*)	1.0	-	3.0
(X_2_) Calcium chloride concentration	% (*w*/*w*)	5.0	-	15.0
(X_3_) Tween 80 ratio	% (*w*/*w*)	5.0	-	15.0
(X_4_) Span 80 ratio	% (*w*/*w*)	5.0	-	15.0
(X_5_) Alginate type	-	Low	Medium	High
**Response**	**Unit**	**Criteria Goals**		
(Y_1_) Alginate core size	µm	In range [below 30 µm]		
(Y_2_) Loading capacity	% (*w*/*w*)	Maximize		
(Y_3_) Encapsulation efficiency	% (*w*/*w*)	In range [upper 50%]		
(Y_4_) The release rate after 1 h	% (*w*/*w*)	In range [below 60%]		

**Table 2:** Experimental space of I-optimal design and the value of the responses.

Run	X_1_ (%)	X_2_ (%)	X_3_ (%)	X_4_ (%)	X_5_	Y_1_ (µm)	Y_2_ (%)	Y_3_ (%)	Y_4_ (%)
1	1.71	6.70	10.98	14.40	Medium	50.1 ± 14.8	18.7 ± 0.4	45.8 ± 1.0	53.4 ± 5.5
2	1.00	14.80	15.00	5.00	High	15.1 ± 5.0	23.5 ± 1.0	41.2 ± 1.7	74.4 ± 2.4
3	2.49	10.45	15.00	15.00	Low	45.6 ± 11.0	16.6 ± 0.9	52.2 ± 2.9	52.6 ± 10.3
4	1.67	7.25	5.05	9.25	Medium	18.9 ± 5.8	23.9 ± 0.4	53.7 ± 0.9	64.9 ± 2.3
5	3.00	7.30	11.20	9.00	Medium	89.7 ± 15.7	2.6 ± 0.1	10.2 ± 0.4	99.5 ± 2.7
6	1.00	5.00	15.00	5.00	Low	12.3 ± 3.8	24.9 ± 0.8	48.5 ± 1.5	81.2 ± 4.8
7	2.90	10.50	5.00	9.85	Medium	45.9 ± 10.6	17.9 ± 0.1	51.9 ± 0.2	78.7 ± 0.2
8	2.43	5.00	7.50	11.05	Low	22.0 ± 5.6	13.0 ± 0.8	41.0 ± 2.4	73.8 ± 2.5
9	1.58	15.00	12.45	9.00	Low	9.7 ± 2.6	21.7 ± 0.6	51.0 ± 1.5	86.9 ± 0.8
10	1.97	14.70	10.00	15.00	Medium	32.2 ± 9.6	13.6 ± 0.6	35.3 ± 1.5	72.8 ± 4.0
11	2.50	7.00	15.00	7.70	High	54.3 ± 14.7	2.2 ± 0.1	7.0 ± 0.2	98.9 ± 0.5
12	3.00	15.00	15.00	5.00	Medium	45.5 ± 10.4	6.3 ± 0.6	25.9 ± 2.6	96.0 ± 0.7
13	3.00	10.25	10.95	5.00	Low	25.1 ± 6.3	10.4 ± 0.4	36.8 ± 1.3	80.4 ± 0.1
14	2.43	5.00	7.50	11.05	Low	28.8 ± 6.5	13.5 ± 0.7	41.8 ± 2.1	74.9 ± 7.3
15	2.40	8.17	6.75	15.00	High	57.5 ± 15.2	12.4 ± 0.5	37.3 ± 1.5	84.8 ± 1.2
16	3.00	5.00	13.56	15.00	Medium	71.5 ± 15.1	6.7 ± 0.5	31.1 ± 2.5	92.9 ± 3.0
17	1.00	15.00	5.00	14.50	High	19.7 ± 6.5	35.3 ± 0.8	63.5 ± 1.4	84.7 ± 1.1
18	1.00	9.80	9.00	15.00	Low	12.8 ± 5.0	30.9 ± 1.0	43.3 ± 1.4	70.9 ± 10.2
19	1.50	9.50	5.00	5.00	Low	19.1 ± 5.5	18.3 ± 0.4	38.5 ± 0.9	59 ± 7.6
20	1.22	12.65	5.00	8.37	High	10.6 ± 3.5	16.7 ± 0.7	28.4 ± 1.2	78.2 ± 11.5
21	1.00	15.00	15.00	14.85	High	11.7 ± 4.2	15.1 ± 0.3	18.1 ± 0.3	77.2 ± 2.5
22	1.58	15.00	12.45	9.00	Low	10.5 ± 3.9	21.6 ± 0.9	51.8 ± 2.1	88.0 ± 4.2
23	3.00	5.00	5.00	5.00	High	70.4 ± 15.0	4.0 ± 0.1	13.3 ± 0.5	92.6 ± 4.0
24	1.07	9.50	15.00	10.10	Medium	54.2 ± 17.4	20.0 ± 1.0	32.1 ± 1.6	73.8 ± 4.1
25	1.00	5.00	15.00	15.00	High	15.8 ± 5.6	14.2 ± 0.3	15.0 ± 0.3	81.7 ± 3.9
26	2.62	12.29	14.95	10.89	Medium	54.9 ± 14.2	6.9 ± 0.4	25.0 ± 1.3	77.5 ± 4.2
27	3.00	15.00	13.00	13.65	High	107.0 ± 16.1	3.9 ± 0.2	13.2 ± 0.7	90.8 ± 7.2
28	1.00	7.00	8.45	8.05	High	11.4 ± 4.0	24.3 ± 0.6	28.0 ± 0.7	73.0 ± 5.1
29	2.40	8.17	6.75	15.00	High	40.8 ± 10.0	12.3 ± 0.3	38.8 ± 0.8	87.1 ± 3.2
30	1.00	5.00	5.00	15.00	Medium	21.4 ± 6.5	14.7 ± 0.4	16.9 ± 0.5	92.8 ± 4.0
31	2.00	5.35	10.00	5.00	Medium	67.0 ± 16.1	12.0 ± 0.8	30.7 ± 2.1	89.1 ± 7.3
32	2.50	7.00	15.00	7.70	High	81.7 ± 17.1	2.6 ± 0.2	8.0 ± 0.5	99.2 ± 8.0
33	3.00	15.00	5.00	15.00	Low	49.5 ± 12.7	10.9 ± 0.7	37.7 ± 2.4	86.1 ± 11.0
34	2.33	14.83	6.78	6.00	High	43.9 ± 13.8	16.1 ± 0.2	52.3 ± 0.7	81.8 ± 2.3
35	1.00	15.00	6.30	5.00	Medium	15.7 ± 4.7	33.4 ± 0.9	55.1 ± 1.4	77.4 ± 3.6
36	1.00	7.00	8.45	8.05	High	14.9 ± 4.6	24.8 ± 0.6	26.0 ± 0.6	72.0 ± 0.3

(X_1_) alginate concentration, (X_2_) calcium chloride concentration, (X_3_) Tween 80 ratio, (X_4_) Span 80 ratio, and (X_5_) alginate type; (Y_1_) alginate core size, (Y_2_) loading capacity, (Y_3_) encapsulation efficiency, and (Y_4_) the in vitro release rate after 1 h. Data were presented as mean ± SD (n = 3).

**Table 3:** Analysis of the effect of factors on the responses by linear regression.

Response	Transform	Model	R^2^	F-Value	*p*-Value
(Y_1_) Alginate core size	Square root	2FI	0.9324	10.34	<0.0001
(Y_2_) Loading capacity	Inverse square root	2FI	0.8936	6.30	0.0004
(Y_3_) Encapsulation efficiency	Natural lograrit	Linear	0.5141	5.11	0.0011
(Y_4_) The in vitro release rate after 1 h	Power (λ = 3)	Quadratic	0.8950	3.91	0.0112

(2FI) 2-factor interaction.

**Table 4:** ANOVA for proposed mathematic model.

Source	Sum of Square	Degrees of Freedom	Mean Square	F-Value	*p*-Value
(Y_1_) Alginate core size					
Model	137.27	20	6.86	10.34	<0.0001
X_1_	64.97	1	64.97	97.88	<0.0001
X_3_	5.82	1	5.82	8.77	0.0097
X_5_	22.49	2	11.25	16.94	0.0001
X_1_.X_5_	12.91	2	6.45	9.72	0.0020
CV%	14.05				
(Y_2_) Loading capacity					
Model	0.54	20	0.03	6.30	0.0004
X_1_	0.21	1	0.21	50.08	<0.0001
X_3_	0.06	1	0.06	14.37	0.0018
X_5_	0.06	2	0.03	7.12	0.0067
X_1_.X_3_	0.03	1	0.03	8.11	0.0122
X_2_.X_4_	0.02	1	0.02	4.59	0.0489
X_3_.X_5_	0.05	2	0.02	5.42	0.0169
CV%	21.80				
(Y_3_) Encapsulation efficiency					
Model	5.88	6	0.98	5.15	0.0010
X_1_	0.89	1	0.89	4.66	0.0394
X_2_	1.13	1	1.13	5.93	0.0213
X_3_	1.36	1	1.36	7.16	0.0121
X_5_	2.88	2	1.44	7.58	0.0023
CV%	47.32				
(Y_4_) The in vitro release rate after 1 h					
Model	1.53 × 10^12^	24	6.38 × 10^10^	3.91	0.0112
X_1_	3.41 × 10^11^	1	3.41 × 10^11^	20.88	0.0008
X_1_.X_5_	1.31 × 10^11^	2	6.57 × 10^10^	4.02	0.0489
X_2_.X_5_	1.84 × 10^11^	2	9.21 × 10^10^	5.64	0.0206
X_3_.X_4_	2.14 × 10^11^	1	2.14 × 10^11^	13.08	0.0041
(X_2_)^2^	2.04 × 10^11^	1	2.04 × 10^11^	12.47	0.0047
CV%	23.09				

(X_1_) alginate concentration, (X_2_) calcium chloride concentration, (X_3_) Tween 80 ratio, (X_4_) Span 80 ratio, (X_5_) alginate type, (CV%) coefficient of variation.

**Table 5:** Optimized formulation suggestions and prediction by Design Expert. Data were presented as mean ± SD (n = 3).

Factor/Response	Design Experts Suggestions	Design Experts Predictions	Experimental Results
(X_1_) Alginate concentration	1.17%	-	
(X_2_) Calcium chloride concentration	7.60%	-	
(X_3_) Tween 80 ratio	5.78%	-	
(X_4_) Span 80 ratio	5.00%	-	
(X_5_) Alginate type	Low viscosity	-	
(Y_1_) Alginate core size	-	11.97 µm	7.70 ± 1.45 µm (*p* = 0.1253)
(Y_2_) Loading capacity	-	24.49%	24.44 ± 0.32% (*p* = 0.8837)
(Y_3_) Encapsulation efficiency	-	61.45%	61.53 ± 0.19% (*p* = 0.8834)
(Y_4_) The in vitro release rate after 1 h	-	57.67%	57.59 ± 0.57% (*p* = 0.9156)

**Table 6:** Analysis of microspheres release kinetics at different pH buffers. Data were presented as mean ± SD (n = 3).

Kinetic Models	pH 7.4		pH 1.2 and 6.8	
	R^2^	K	R^2^	K
Zero-order	0.3543	16.56 ± 0.09	0.1511	6.26 ± 0.06
First-order	0.8577	0.51 ± 0.01	0.9957	0.17 ± 0.00
Higuchi	0.9264	38.32 ± 0.14	0.9086	23.81 ± 0.19
Hixson–Crowell	0.7601	0.13 ± 0.00	0.9915	0.05 ± 0.00
Korsmeyer–Peppas	0.9937	47.45 ± 0.55 n = 0.35 ± 0.01	0.9241	28.00 ± 0.01 n = 0.43 ± 0.00
Peppas–Sahlin	0.9937	K_1_ = 47.59 ± 4.53 K_2_ = −0.10 ± 3.94 m = 0.35 ± 0.03	0.9960	K_1_ = 17.80 ± 0.70 K_2_ = −0.78 ± 0.06 m = 0.83 ± 0.02

**Table 7:** Effect of vitexin–isovitexin-loaded microspheres on blood glucose levels in alloxan-induced hyperglycemic mice.

Group	Day 1	Day 7	Day 14	Day 21
Control	120.571 ± 4.001	86.071 ± 5.073	100.000 ± 1.674	109.286 ± 7.196
ALX	287.857 ± 21.122 ****	270.429 ± 43.562 ***	328.143 ± 34.149 ****	453.571 ± 26.977 ****
GBC	273.667 ± 23.369 ****	314.333 ± 40.470 ****	278.333 ± 33.830 **	275.000 ± 49.592 **^,###^
mVTX/iVTX 30	295.857 ± 21.641 ****	311.143 ± 31.982 ***	331.143 ± 30.237 ****	281.429 ± 41.212 **^,##^
mVTX/iVTX 60	271.000 ± 14.260 ****	272.375 ± 33.405 **	316.625 ± 34.271 ****	177.000 ± 35.982 ^####^

Data were presented as mean ± S.E.M. (n = 7–14). Comparisons between the groups were carried out by a one-way analysis of variance (ANOVA), followed by a Tukey post hoc test. ** *p* < 0.01, *** *p* < 0.001, **** *p* < 0.0001 vs. control group. ^##^ *p* < 0.01, ^###^ *p* < 0.001, ^####^ *p* < 0.0001 vs. ALX group. ALX = alloxan, GBC = glibenclamide, and mVTX/iVTX = vitexin–isovitexin-loaded microspheres.

**Table 8:** AUC of blood glucose levels in alloxan-induced hyperglycemic mice.

Time (Days)	1–7	1–14	1–21
Control	619.929 ± 18.111	1271.179 ± 37.777	2003.679 ± 59.679
ALX	1674.857 ± 184.954 ****	3769.857 ± 432.573 ****	6505.857 ± 575.539 ****
GBC	1764.000 ± 181.351 ****	3838.333 ± 368.046 ****	5775.000 ± 545.439 ****
mVTX/iVTX 30	1821.000 ± 155.659 ****	4069.000 ± 361.217 ****	6213.000 ± 564.951 ****
mVTX/iVTX 60	1630.125 ± 116.547 ****	3691.625 ± 325.562 ****	5419.313 ± 466.729 ****

Data were presented as mean ± S.E.M. (n = 7–14). Comparisons between the groups were carried out by a one-way analysis of variance (ANOVA), followed by a Tukey post hoc test. **** *p* < 0.0001 vs. control group. ALX = alloxan, GBC = glibenclamide, and mVTX/iVTX = vitexin–isovitexin-loaded microspheres.

**Table 9:** AUC of blood glucose levels in oral glucose tolerance test.

Time (mins)	0–15	0–30	0–60	0–120
Glucose	2642.81 ± 98.21	6310.31 ± 382.02	11,560.31 ± 927.79	17,534.06 ± 1261.74
GBC	1668.75 ± 144.46 ****	3415.31 ± 333.74 ****	5817.19 ± 562.26 ****	8963.44 ± 743.66 ****
mVTX/iVTX 30	2197.50 ± 174.02	4864.69 ± 330.71 **^,##^	9017.81 ± 517.87 *^,##^	15,385.31 ± 859.79 ^####^
mVTX/iVTX 60	1989.38 ± 69.24 **	4216.88 ± 164.65 ****	7657.50 ± 256.44 ***	13,781.25 ± 397.35 *^,##^

Data were presented as mean ± S.E.M. (n = 8). Comparisons between the groups were carried out by a one-way analysis of variance (ANOVA), followed by a Tukey post hoc test. * *p* < 0.05, ** *p* < 0.01, *** *p* < 0.001, **** *p* < 0.0001 vs. glucose group. ^##^ *p* < 0.01, ^####^ *p* < 0.0001 vs. GBC group. GBC = glibenclamide, and mVTX/iVTX = vitexin–isovitexin-loaded microspheres.

**Table 10:** Effect of vitexin–isovitexin-loaded microspheres on HbA1c and insulin levels.

Group	HbA1c (%)	Insulin (μU/mL)
Control	4.367 ± 0.110	0.169 ± 0.029
ALX	7.715 ± 0.166 ****	0.123 ± 0.017
GBC	6.600 ± 0.352 ****	0.137 ± 0.018
mVTX/iVTX 30	6.994 ± 0.478 ****	0.173 ± 0.037
mVTX/iVTX 60	6.725 ± 0.504 ****	0.150 ± 0.012

Data were presented as mean ± S.E.M. (n = 7–14). Comparisons between the groups were carried out by a one-way analysis of variance (ANOVA), followed by a Tukey post hoc test. **** *p* < 0.0001 vs. control group. ALX = alloxan, GBC = glibenclamide, and mVTX/iVTX = vitexin–isovitexin-loaded microspheres.

**Table 11:** Effect of vitexin–isovitexin-loaded microspheres on HOMA-IR and HOMA-β indices.

Group	HOMA-IR	HOMA-β
Control	0.0457 ± 0.0088	1.2213 ± 0.2784
ALX	0.1277 ± 0.0163 **	0.1137 ± 0.0296
GBC	0.0995 ± 0.0221	0.3878 ± 0.1417
mVTX/iVTX 30	0.1103 ± 0.0399	0.3820 ± 0.0395
mVTX/iVTX 60	0.1006 ± 0.0139	0.3088 ± 0.1171

Data were presented as mean ± S.E.M. (n = 7–14). Comparisons between the groups were carried out by a one-way analysis of variance (ANOVA), followed by a Tukey post hoc test. ** *p* < 0.01 vs. control group. ALX = alloxan, GBC = glibenclamide, and mVTX/iVTX = vitexin–isovitexin-loaded microspheres.

## Data Availability

The data presented in this study are available upon request from the corresponding author.

## Supplementary Materials

The following supporting information can be downloaded at: https://www.mdpi.com/article/10.3390/pharmaceutics17070819/s1, Table S1. Procedure in α-glucosidase inhibitory activity test. Table S2. The full ANOVA analysis. Table S3. Effect of vitexin–isovitexin-loaded microspheres on ALT levels in alloxan-induced hyperglycemic mice. Data were presented as mean ± S.E.M. (n = 7–14). Comparisons between the groups were carried out by a one-way analysis of variance (ANOVA), followed by a Tukey post hoc test. ALX = alloxan, GBC = glibenclamide, and mVTX/iVTX = vitexin–isovitexin-loaded microspheres. Figure S1. α-glucosidase inhibitory activity of vitexin–isovitexin. Figure S2. α-glucosidase inhibitory activity of acarbose. Figure S3. The body weight of mice in 14 days. Data were presented as mean ± S.E.M. (n = 6). Comparisons between the groups were carried out by a one-way analysis of variance (ANOVA), followed by a Tukey post hoc test. VTX/iVTX = vitexin–isovitexin. The authors have cited additional references within the Supplementary Materials [96,97].

## Abbreviations

The following abbreviations are used in this manuscript:

T_1/2_	Half-life
2FI	Two-factor interaction
ALX	Alloxan
ANOVA	One-way analysis of variance
AUC	Area under the curve
CV%	Coefficient of variation
DSC	Differential scanning calorimetry
EE%	Encapsulation efficiency
GBC	Glibenclamide
H&E	Hematoxylin and Eosin
HOMA	Homeostatic model assessment
HPLC	High-performance liquid chromatography
IR	Insulin resistance
LC	Loading capacity
mVTX/iVTX	Vitexin–isovitexin-loaded microspheres
OGTT	Oral glucose tolerance test
PECs	Polyelectrolyte complexes
rpm	Revolutions per minute
S.E.M.	Standard error of mean
SEM	Scanning electron microscopy
W/O	Water-in-oil
*w*/*w*	Weight/weight
