# Supplementary material for: Optimization of Controlled-Release Microspheres Containing Vitexin and Isovitexin Through Experimental Design and Evaluation of Their Hypoglycemic Effects
Source: Pharmaceutics. 2025 Jun 24;17(7):819. doi: 10.3390/pharmaceutics17070819 (PMC12299104; doi:10.3390/pharmaceutics17070819)
Supplement: Supplementary file 1 [file pharmaceutics-17-00819-s001.zip › pharmaceutics-3682766-supplementary.pdf]

SUPPLEMENTARY METHODS

1. α-glucosidase inhibitory activity of vitexin-isovitexin

The α-glucosidase inhibitory activity was evaluated by measuring the absorbance at 405 nm using p-NPG as the substrate, performed in a 96-well plate following the procedures described by Guzmán *et al*. [1] and Wang *et al*. [2], with slight modifications.

150 µL of phosphate buffer (pH 6.8) was added to each well. 10 µL of test sample dissolved in DMSO at different concentrations were added. Then, 10 µL of 0.1 U/mL α-glucosidase solution was added. The mixture was incubated for 15 min at 25 °C. 30 µL of 2 mM p-NPG solution was added, and the mixture was further incubated for 30 min at 37 °C. The absorbance was measured at 405 nm every minute for 15 min.

The control sample used 10 µL of DMSO instead of the test sample. The blank sample used 10 µL of buffer instead of the α-glucosidase solution. Acarbose was used as the reference compound.

Supplementary Table S1. Procedure in α-glucosidase inhibitory activity test.

Sample solutions	Test sample	Test control	Blank sample	Blank control
**Potassium phosphate buffer pH 6.8**	150 µL	160 µL	150 µL	160 µL
**α-glucosidase 0.1 U/mL**	10 µL	-	10µL	-
**DMSO**	-	-	10 µL	10 µL
**Inhibitor compound**	10 µL	10 µL	-	-
	**Incubate at 25 °C for 15 min**			
**p-NPG 2 mM**	30 µL	30 µL	30 µL	30 µL
	**Mix well for 10 sec, measure absorbance at 405 nm every 1 min for 15 min**			

The inhibition percentage (I%) of the test samples was calculated using the formula:

$$I\% = (1 - \frac{v_s}{v_c}) \times 100\%$$

Where v_s is the velocity of the test sample and v_c is the velocity of the control.

The IC_{50} values of the inhibitory compounds were determined using nonlinear regression analysis performed on GraphPad Prism 9 software. The results are presented in the form of a sigmoidal curve, with the IC_{50} value expressed as the mean ± S.E.M.

2. Acute toxicity of vitexin-isovitexin

The acute toxicity test was conducted on Swiss albino mice following OECD 420 guidelines [3]. Mice were randomly divided into 4 groups as follows:

Control group: Distilled water, p.o.

VTX/iVTX 2 group: Vitexin-isovitexin 2 g/kg, p.o.

VTX/iVTX 5 group: Vitexin-isovitexin 5 g/kg, p.o.

VTX/iVTX 10 group: Vitexin-isovitexin 10 g/kg, p.o.

Mice were fasted overnight and subsequently administered a single dose according to the treatment regimens mentioned above. The animals were observed for 14 days to record mortality rates and any potential signs of toxicity. Body weight was recorded daily.

3. Effect of vitexin-isovitexin-loaded microspheres on alanine aminotransferase (ALT) level in alloxan-induced hyperglycemic mice

The assay of ALT was carried out using the ALT/GPT 4+1 SL reagent (ALSL-0430, ELITech Group, France) according to the guidelines of the manufacturer. Briefly, the reagent and sample were mixed and incubated for 1 min and measured at 340 nm at 37 °C. The ALT level was calculated as follows:

$$\frac{\Delta A}{min.} \times 3333$$

Unit of ALT: IU/L

SUPPLEMENTARY RESULTS

1. Full ANOVA analysis for regression mathematic model

In the optimization, the ANOVA analysis was applied to all the response obtained to study the significance of the model. **Supplementary Table S2** showed detail regression coefficient for the suitable transform and significance probability (P-value) of each factor.

Supplementary Table S2. The full ANOVA analysis.

Source	Sum of Squares	df	Mean Square	F-value	p-value	
(Y_1) Alginate core size						
Transform: Square Root						
**Model**	137.27	20	6.86	10.34	< 0.0001	significant
**X_1**	64.97	1	64.97	97.88	< 0.0001	
**X_2**	2.33	1	2.33	3.52	0.0804	
**X_3**	5.82	1	5.82	8.77	0.0097	
**X_4**	0.3238	1	0.3238	0.4878	0.4956	
**X_5**	22.49	2	11.25	16.94	0.0001	
**X_1X_2**	0.1131	1	0.1131	0.1704	0.6856	
**X_1X_3**	0.0111	1	0.0111	0.0168	0.8987	
**X_1X_4**	0.4175	1	0.4175	0.6289	0.4401	
**X_1X_5**	12.91	2	6.45	9.72	0.0020	
**X_2X_3**	1.53	1	1.53	2.30	0.1501	
**X_2X_4**	2.46	1	2.46	3.70	0.0735	
**X_2X_5**	4.79	2	2.39	3.61	0.0526	
**X_3X_4**	0.0427	1	0.0427	0.0643	0.8032	
**X_3X_5**	3.92	2	1.96	2.95	0.0829	
**X_4X_5**	2.09	2	1.05	1.58	0.2391	
**Residual**	9.96	15	0.6638			
(Y_2) Drug loading						
Transform: Inverse Sqrt						
**Model**	0.5389	20	0.0269	6.30	0.0004	significant
**X_1**	0.2143	1	0.2143	50.08	< 0.0001	
**X_2**	0.0158	1	0.0158	3.70	0.0736	
**X_3**	0.0615	1	0.0615	14.37	0.0018	
**X_4**	0.0065	1	0.0065	1.52	0.2370	
**X_5**	0.0610	2	0.0305	7.12	0.0067	
**X_1X_2**	0.0093	1	0.0093	2.17	0.1618	
**X_1X_3**	0.0347	1	0.0347	8.11	0.0122	
**X_1X_4**	0.0082	1	0.0082	1.93	0.1853	
**X_1X_5**	0.0250	2	0.0125	2.92	0.0847	
**X_2X_3**	0.0042	1	0.0042	0.9792	0.3381	
**X_2X_4**	0.0196	1	0.0196	4.59	0.0489	
**X_2X_5**	0.0068	2	0.0034	0.7941	0.4701	

**X_3X_4**	0.0047	1	0.0047	1.10	0.3110	
**X_3X_5**	0.0464	2	0.0232	5.42	0.0169	
**X_4X_5**	0.0034	2	0.0017	0.3988	0.6780	
**Residual**	0.0642	15	0.0043			
(Y_3) In vitro release rate in 1 hour						
Transform: Power, Lambda: 3						
**Model**	1.532E+12	24	6.384E+10	3.91	0.0112	significant
**X_1**	3.411E+11	1	3.411E+11	20.88	0.0008	
**X_2**	5.692E+09	1	5.692E+09	0.3483	0.5670	
**X_3**	1.895E+10	1	1.895E+10	1.16	0.3046	
**X_4**	2.532E+10	1	2.532E+10	1.55	0.2391	
**X_5**	1.228E+11	2	6.138E+10	3.76	0.0571	
**X_1X_2**	4.884E+10	1	4.884E+10	2.99	0.1118	
**X_1X_3**	5.534E+10	1	5.534E+10	3.39	0.0928	
**X_1X_4**	3.927E+10	1	3.927E+10	2.40	0.1494	
**X_1X_5**	1.314E+11	2	6.568E+10	4.02	0.0489	
**X_2X_3**	3.040E+10	1	3.040E+10	1.86	0.1998	
**X_2X_4**	1.819E+05	1	1.819E+05	0.0000	0.9974	
**X_2X_5**	1.842E+11	2	9.210E+10	5.64	0.0206	
**X_3X_4**	2.137E+11	1	2.137E+11	13.08	0.0041	
**X_3X_5**	9.718E+09	2	4.859E+09	0.2974	0.7486	
**X_4X_5**	4.565E+10	2	2.283E+10	1.40	0.2880	
**$(X_1)^2$**	7.626E+10	1	7.626E+10	4.67	0.0537	
**$(X_2)^2$**	2.038E+11	1	2.038E+11	12.47	0.0047	
**$(X_3)^2$**	8.526E+08	1	8.526E+08	0.0522	0.8235	
**$(X_4)^2$**	3.122E+10	1	3.122E+10	1.91	0.1943	
**Residual**	1.797E+11	11	1.634E+10			
(Y_4) Encapsulation efficiency						
Transform: Natural Log						
**Model**	5.86	6	0.9770	5.11	0.0011	significant
**X_1**	0.8804	1	0.8804	4.61	0.0403	
**X_2**	1.12	1	1.12	5.89	0.0217	
**X_3**	1.37	1	1.37	7.17	0.0121	
**X_4**	0.0167	1	0.0167	0.0877	0.7693	
**X_5**	2.87	2	1.43	7.50	0.0024	
**Residual**	5.54	29	0.1910			

Notes: (X_1) alginate concentration, (X_2) calcium chloride concentration, (X_3) Tween 80 ratio, (X_4) Span 80 ratio, (X_5) alginate type, (Y_1) alginate core size, (Y_2) loading capacity, (Y_3) encapsulation efficiency, and (Y_4) the in vitro release rate after 1h.

2. *In vitro* vitexin-isovitexin release study

The controlled release of vitexin-isovitexin from the microspheres (shown in Supplementary Table S3) was sustained up to 8 h in pH 7.4 and extended to 24 h in the digestive medium (pH 1.2 and 6.8). In comparison, the optimized alginate cores exhibited a rapid release profile. The alginate-chitosan microspheres demonstrated a more controlled release profile.

Supplementary Table S3. The *in vitro* release data of the alginate cores, alginate-chitosan microspheres in different pH buffers. Data were presented as mean ± SD (n = 3).

pH 7.4			pH 1.2 and 6.8		
**Time (hour)**	**Alginate cores**	**Microspheres**	**Time(hour)**	**Alginate cores**	**Microspheres**
0	0.00 ± 0.00	0.00 ± 0.00	0 (pH 1.2)	0.00 ± 0.00	0.00 ± 0.00
0.25	33.61 ± 3.47	27.30 ± 0.26	1 (pH 1.2)	32.06 ± 2.45	15.96 ± 0.71
0.50	38.98 ± 1.41	35.98 ± 0.95	2 (pH 1.2)	41.93 ± 2.30	25.10 ± 1.23
0.75	48.20 ± 0.93	43.19 ± 1.74	3 (pH 6.8)	62.36 ± 1.16	42.88 ± 1.45
1	57.59 ± 0.57	52.80 ± 1.62	4 (pH 6.8)	71.96 ± 1.69	49.50 ±1.86
2	70.99 ± 1.66	61.20 ± 1.62	5 (pH 6.8)	83.39 ± 2.64	58.24 ±1.58
3	76.13 ± 2.98	66.60 ± 1.19	6 (pH 6.8)	87.84 ± 3.22	62.42 ± 0.62
4	82.30 ± 0.19	75.95 ± 0.61	7 (pH 6.8)	94.54 ± 2.15	70.50 ± 0.43
5	90.39 ± 0.98	81.01 ± 0.95	8 (pH 6.8)	94.93 ± 3.34	73.95 ± 1.06
6	99.93 ± 0.73	87.52 ± 1.02	9 (pH 6.8)	97.00 ± 1.12	79.83 ± 1.07
8	100.26 ± 1.29	95.19 ± 1.27	10 (pH 6.8)	101.55 ± 0.67	84.60 ± 1.24
			12 (pH 6.8)		87.16 ± 1.00
			16 (pH 6.8)		91.46 ± 1.13
			20 (pH 6.8)		97.03 ± 1.79
			24 (pH 6.8)		99.53 ± 1.94

3. α-glucosidase inhibitory activity of vitexin-isovitexin

The α-glucosidase inhibitory activity of vitexin-isovitexin and acarbose were evaluated across a range of appropriate concentrations (**Supplementary Fig. S1 and S2**). The results demonstrated a notable inhibitory effect on α-glucosidase activity in a concentration-dependent manner, with the observed IC_{50} values of vitexin-isovitexin and acarbose were 1.47 ± 0.472 mM and 0.13 ± 0.01 mM, respectively.

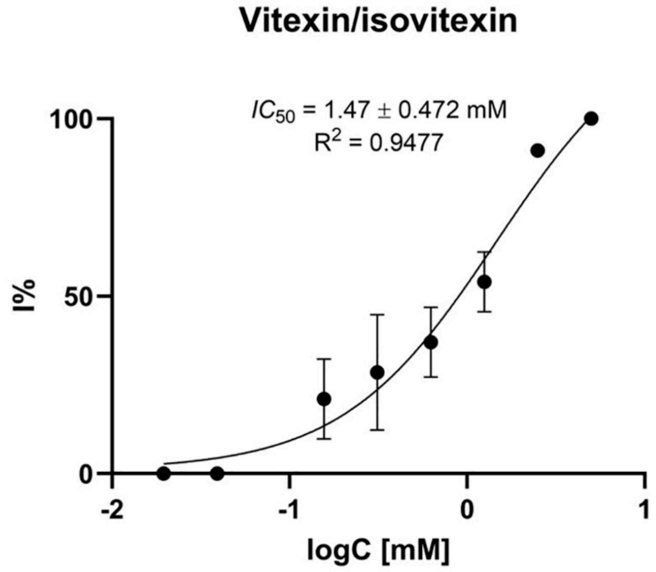

Supplementary Fig. S1. α-glucosidase inhibitory activity of vitexin-isovitexin.

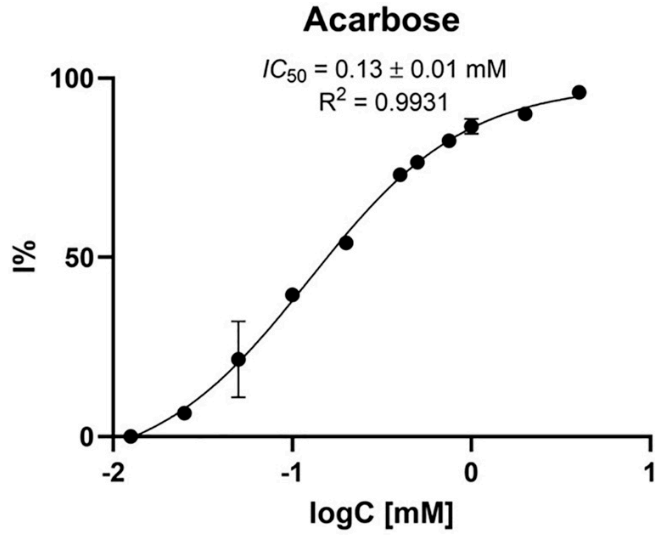

Supplementary Fig. S2. α-glucosidase inhibitory activity of acarbose.

4. Acute toxicity of vitexin-isovitexin

During 14 days, there was no mortality in mice treated with three doses of vitexin-isovitexin. In addition, mice treated with vitexin-isovitexin also showed no significant changes in appearance, behavioral patterns, water and food consumption when compared with mice in the control group. At day 14, all three vitexin-isovitexin groups had approximately equivalent body weights compared to the control group.

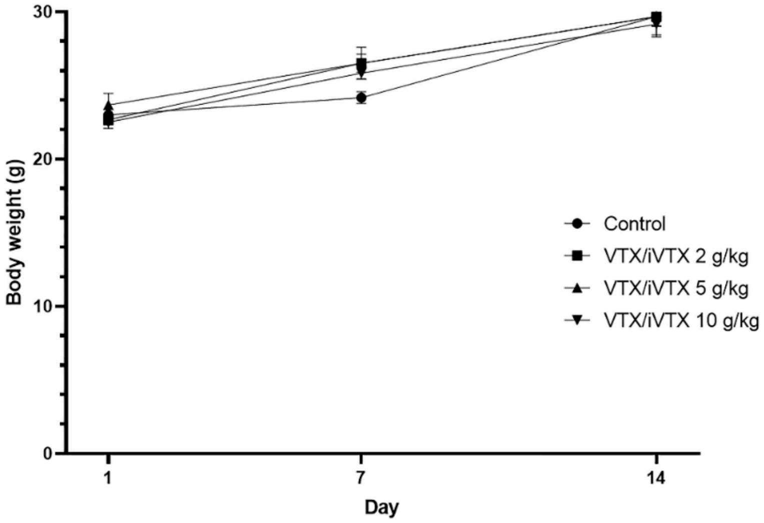

Supplementary Fig. S3. The body weight of mice in 14 days. Data were presented as mean ± S.E.M. (n = 6). Comparisons between the groups were made by one-way analysis of variance (ANOVA), followed by a Tukey post-hoc test. VTX/iVTX = vitexin-isovitexin.

5. Effect of vitexin-isovitexin-loaded microspheres on alanine aminotransferase (ALT) level

Supplementary Table S3. Effect of vitexin-isovitexin-loaded microspheres on ALT levels in alloxan-induced hyperglycemic mice. Data were presented as mean ± S.E.M. (n = 7 – 14). Comparisons between the groups were made by one-way analysis of variance (ANOVA), followed by a Tukey post-hoc test. ALX = alloxan, GBC = glibenclamide, and mVTX/iVTX = vitexin-isovitexin-loaded microspheres.

Group	ALT (IU/L)
Control	38.033 ± 3.691
ALX	100.254 ± 31.285
GBC	95.200 ± 35.453
mVTX/iVTX 30	48.900 ± 4.068
mVTX/iVTX 60	65.275 ± 12.992

In terms of ALT level, all treatment groups receiving glibenclamide and vitexin-isovitexin-loaded microspheres had lower ALT levels than that of the untreated group. Two groups administered vitexin-isovitexin showed a better ALT-regulating effect than the GBC group. Notwithstanding, there was no statistically significant difference between all groups.

Supplementary References

[1] G.-G. Graciela, C.-R. Rocio, T. Noemi Waksman de, S.-A. Ricardo, Optimization and Validation of a Microscale In vitro Method to Assess α-Glucosidase Inhibition Activity, Current Analytical Chemistry, 14 (2018) 458-464.

[2] Yin, Z.; Zhang, W.; Feng, F.; Zhang, Y.; Kang, W. α-Glucosidase inhibitors isolated from medicinal plants. Food Sci. Hum. Wellness 2014, 3, 136–174. https://doi.org/10.1016/j.fshw.2014.11.003.

[3] P.-B.T. Guideline, OECD guideline for the testing of chemicals, The Hershberger, 601 (2001) 858.
